# Predicting Grizzly Bear Density in Western North America

**DOI:** 10.1371/journal.pone.0082757

**Published:** 2013-12-18

**Authors:** Garth Mowat, Douglas C. Heard, Carl J. Schwarz

**Affiliations:** 1 Natural Resource Science Section, Ministry of Forests, Lands and Natural Resource Operations, Nelson, British Columbia, Canada; 2 Fish and Wildlife Section, Ministry of Forests, Lands and Natural Resource Operations, Prince George, British Columbia, Canada; 3 Department of Statistics and Actuarial Sciences, Simon Fraser University, Burnaby, British Columbia, Canada; Université de Sherbrooke, Canada

## Abstract

Conservation of grizzly bears (*Ursus arctos*) is often controversial and the disagreement often is focused on the estimates of density used to calculate allowable kill. Many recent estimates of grizzly bear density are now available but field-based estimates will never be available for more than a small portion of hunted populations. Current methods of predicting density in areas of management interest are subjective and untested. Objective methods have been proposed, but these statistical models are so dependent on results from individual study areas that the models do not generalize well. We built regression models to relate grizzly bear density to ultimate measures of ecosystem productivity and mortality for interior and coastal ecosystems in North America. We used 90 measures of grizzly bear density in interior ecosystems, of which 14 were currently known to be unoccupied by grizzly bears. In coastal areas, we used 17 measures of density including 2 unoccupied areas. Our best model for coastal areas included a negative relationship with tree cover and positive relationships with the proportion of salmon in the diet and topographic ruggedness, which was correlated with precipitation. Our best interior model included 3 variables that indexed terrestrial productivity, 1 describing vegetation cover, 2 indices of human use of the landscape and, an index of topographic ruggedness. We used our models to predict current population sizes across Canada and present these as alternatives to current population estimates. Our models predict fewer grizzly bears in British Columbia but more bears in Canada than in the latest status review. These predictions can be used to assess population status, set limits for total human-caused mortality, and for conservation planning, but because our predictions are static, they cannot be used to assess population trend.

## Introduction

Grizzly bear hunting is controversial because of peoples' conflicting values and interests. Sarewitz [1] argued that progress toward solving environmental controversies must come primarily from political processes rather than from scientific research. He regards the idea that scientific facts and theories will help settle disputes or build the appropriate foundations for guiding environmental policy as old-fashioned and suggests that the role of science is to collect information to support the implementation of policies determined through political processes. Where grizzly bear hunting opportunity is managed by a quota system, a maximum allowable kill rate is prescribed (the policy) which is then applied to estimates of population size (the scientific information). This paper describes an approach to predict grizzly bear densities that can be used to support, monitor, and evaluate policy decisions.

In the last 15 years, improvements in aerial survey [2] and genetic identification techniques [3] have led to a proliferation of grizzly bear density estimates [4]. However, due to the high cost and the vast areas involved, field-based density estimates have been and likely will continue to be restricted to a small subset of hunted populations.

Three approaches for predicting grizzly bear density have been proposed where field-based estimates are unavailable. 1. Measures of grizzly bear abundance can be generated by assigning densities based on expert opinion regarding the value of landcover attributes, supported by, or in conjunction with, field estimates derived in similar ecosystems [5]. No expert-based models to date have estimated confidence limits for the resulting density estimates so evaluating conservation risk was subjective. Expert models have not considered fundamental concepts, such as whether microsite attributes sum or scale up to provide an indication of landscape scale density [6]. In spite of their shortcomings, expert-based models have been used in all jurisdictions in Canada where grizzly bear hunting is allowed [5], [7]–[9].

2. Resource selection function models can be used to predict the absolute or relative probability of occurrence [10], [11] and possibly density [12] within small ecological units. Although occurrence models may be statistically explanatory and objective, considerable subjectivity may be required when deciding how and where to apply them (e.g., [10]). Models vary with the local availability of resources and behaviors related to regional life history or human influence, and they do not generalize well to other landscapes [11], [13]–17. This is a considerable problem given that grizzly bears occupy a wide range of environments and have many different life history strategies.

3. Trend data can be used to predict density from areas with known abundance [18]. This method requires relatively precise measures of trend that are independent of variation in bear abundance. These data were provided by moose hunters in a Swedish example [18]. Similar data do not exist in North America and we were hence confined to one of the two modeling approaches.

For the greatest general application, predictive abundance models must be underpinned by an understanding of the functional processes affecting density, use direct measures of resource abundance, and apply to all environments and life history strategies [19]. Recent work has quantified relationships between abundance and landscape scale measures of environmental attributes for white-tailed deer (Odocoileus virginianus; [20]), carnivores [21], tassel-eared squirrels (Sciurus aberti) [22], coyotes (Canis latrans) [23], kangaroos (Macropus spp.) [24] and people [25]. Most of these models included one or more measures of food, indexed either by a direct measure of the resource, such as tree basal area for squirrels, or remote measures, such as temperature, precipitation and soil type for humans.

We considered both bottom–up (food supply) and top-down (competition and predation) influences on grizzly bear abundance. Grizzly bears are omnivores and their reliance on animal protein varies greatly across their range [26]–[28]. The single largest meat source in their diet in coastal areas is spawning salmon and all areas of very high bear density have large numbers of salmon over much of the non-denning season [2], [28]. Many populations have no access to salmon and in the continental interior, grizzly bears eat plants, especially fruit, and supplement their diet with insects, rodents, freshwater fish, and ungulates where available [26]–[28]. Bears prefer highly digestible plant species and parts, because they have a simple stomach and are relatively inefficient at digesting plant matter [29], [30]. Preferred species of plants where bears eat the vegetative structure can be considered hydrophilic and bears in the interior concentrate their foraging in moist sites [27], [31]. The exception being when they are digging for corms or roots of various plants. Fruits of several shrubs are highly preferred in late summer and fall and, along with meat, are the basis for the fat deposition required for winter hibernation [32]. The abundance of grizzly bear foods is not only related to ecosystem productivity but also, for bears that live in forested environments, to successional stage [33].

Competition may limit grizzly bear density where they are sympatric with black bears (Ursus americanus) [34], [35]. Black bears and grizzly bears have similar digestive and foraging efficiencies [30], [36], [37] and any competitive advantage of black bears over grizzly bears feeding on plants and fruits is based largely on the smaller body size of black bears [30], [37]. Where grizzly bears rely on meat, they often appear to exclude black bears from the meat source [2], [38], [39], probably because the meat source is clumped and therefore defendable, unlike plant and fruit supplies. Areas that are largely devoid of trees do not support black bears where their range overlaps that of grizzly bears [2], [26], [34], [39], [40]. Presumably grizzly bears exclude black bears from large open areas because black bears cannot seek refuge from grizzly bear aggression by climbing trees [34], [40], [41]. Historic persecution levels may also influence potential competition between the two bear species and this may be mediated by reproduction in grizzly bears [42]. Reproduction is higher in black bears than grizzly bears [34], [35].

Grizzly bears have no significant predators [43], [44], but social factors may limit bear numbers. For example, female bears may avoid important feeding areas to minimize the chance of male bears killing their offspring [45], [46]. Several different hypotheses have been proposed regarding social regulation in grizzly bears [44], however a recent analysis of field data for 4 sites did not support the importance of any form of social regulation on density [47].

Humans limit grizzly numbers by direct mortality, habitat loss, and displacement due to disturbance. Mortality obviously reduces density temporarily, but the relationship between mortality rate and density is complex due to the effects of age ratios and density dependence on vital rates. Habitat loss, and environmental change that completely precludes occupancy by grizzly bears, obviously reduces density. Disturbance has been shown to reduce grizzly bear density at fine scales (e.g., along road corridors and near developments [48], [49]), but the link between disturbance and landscape scale population density, although largely accepted by practitioners [50], has never been demonstrated empirically. Human density may index the above 3 factors, but the functional link to bear density is unclear.

In this paper we modeled the relationship between existing grizzly bear density estimates and potential limiting factors. We then use those relationships to predict grizzly bear density across a large and varied portion of their Canadian range and demonstrate how the use of these predictions for setting hunting quotas and evaluating past levels of human-caused mortality can support the process of developing grizzly bear conservation policy.

## Materials and Methods

### Model development

Based on previous research we felt that the following factors may functionally influence grizzly bear density at the population scale: plant productivity, vegetation type, fish and meat availability, scramble competition with black bears, human disturbance, and human-caused mortality. Based on this functional model, we began by assembling and deriving indices for these factors while attempting to choose measures that were not highly correlated with other factors to avoid collinearity (Table 1). Variables that described food limitation were: salmon availability, the proportion of salmon or ungulates and other non-plant foods in the diet, remote sensing measures of plant productivity, and the proportion of the landscape covered by trees and herbaceous vegetation. Competition with black bears may be best described by measures of black bear abundance, but these were so few that we used tree cover or simple presence or absence of black bears as surrogates for more direct measures of competition.

**Table 1:** Factors hypothesized to limit grizzly bear density in interior ecosystems in North America and the variables we derived to index these factors.

Plant productivity	Vegetation type^1^	Diet	Competition with black bears	Human disturbance	Human-caused mortality
annual precipitation	tree10	salmon presence	tree10	tree10	human density
annual temperature	tree25	%salmon in diet	tree25	tree25	human+livestock density
annualized NDVI	herb50	%terrestrial meat in diet	black bear presence	livestock density	human+livestock density within 10 km^2^
evapotranspiration	herb75			human density	human+livestock density within 50 km^2^
ruggedness	herb100			human+livestock density	mean recorded human-caused mortality in past 10 years
				human+livestock density within 10 km^2^	
				human+livestock density within 50 km^2^	

Appendix S1 for detailed description of GIS derived variables. We digitized the study area boundary for each study area and calculated the average for each index using a GIS. See Table S1 in

=  the proportion of the study with pixels rated as >50% herb/shrub.^1^ This is the sum of all pixels with >the stated percentage of described cover. For example, herb50

^2^ This is the mean human and livestock (sheep and cattle) density (summed) for the area within 10 or 50 km of the study area boundary.

The only mortality factor we considered was direct killing by people. Although it has been mandatory to report all human-caused deaths in all jurisdictions in North America since the 1970's, a substantial proportion of that mortality goes unreported [51] including traditional use by First Nations. The direct effect of human-caused habitat loss in urban areas was accounted for in the remote sensing vegetation measures above. We used human and livestock census information as measures of both the loss of habitat effectiveness, due to behavioral decisions of bears to avoid areas frequented by humans and, the impact of unrecorded human-caused mortality. Other surrogates, such as road density or the number or proportion of problem grizzly bears killed, were difficult to standardize across jurisdictions and were more temporally variable. We did not consider predation, diseases, parasites, or social limitation, because there is no evidence that those were general limiting factors. Hence our three over-arching hypotheses were based on food limitation, scramble competition with black bears, and human limits to density via mortality, habitat loss, and decreased habitat effectiveness.

### The dependent variable - meta analysis of grizzly bear abundance

We critically reviewed estimates of grizzly bear population size or density in the published and unpublished literature. We were interested in estimates for landscapes large enough to represent a grizzly bear population affected largely by births and deaths rather than immigration and emigration, so we only used data where study area size was >800 km^2^ (range 789–22,875 km^2^) or approximately 10 female home ranges, and contained at least 15 resident bears (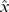 = 80, range 15–765). We used all population estimates that met the above criteria and where we judged the authors had done enough field sampling to generate an estimate that was indicative of the ecosystem. We noted whether authors accounted for bears that were not detected during fieldwork, which generally required the use of mark-recapture analysis, but we accepted more subjective assessments for some intensive census studies. We also noted whether authors had accounted for closure bias, which is the positive bias caused by movement in and out of the study area during the study. We indexed the accuracy of each population estimate by standardizing the confidence limit (CL) width as a percent of the point estimate so we could weight each observation in the analysis. We arbitrarily doubled the width of the reported upper confidence limit (UCL) if authors did not consider incomplete detection (3 areas) or the lower confidence limit (LCL) if authors did not account for closure bias (2 areas). Where no measure of precision was given, we assigned CLs based on survey effort. We arbitrarily assigned the LCL as 50% of the point estimate, but if the authors considered closure bias we reduced the LCL to 25% of the estimate. We doubled the point estimate to index the UCL unless the authors accounted for incomplete detection explicitly, in which case we reduced the UCL to 50% of the point estimate. We created confidence limits using the above ad-hoc rules in 52 of 109 observations including all 16 unoccupied areas discussed below and these were larger than most probability based limits.

We selected 16 study areas in places that were historically occupied by grizzly bears, but were currently not occupied. These areas were all adjacent to occupied areas and there was no known barrier to dispersal. We chose these areas to represent the range of contemporary forces that work to exclude grizzly bears from parts of their range. We selected areas of similar size to other studies in those ecosystems and derived measures for independent variables as for occupied study areas. Though the density estimate was zero, based on local knowledge, we assigned upper CLs based on trapping results, non-hunter kills and the recent record of bear sightings in the study area.

We revised all grizzly bear density estimates by removing the area of water, rock, and bare ground, because we considered these unsuitable to bears.

### Independent variables - derivation of surrogates for limiting factors

We derived average annual precipitation, average annual temperature, NDVI, and evapotranspiration to index plant productivity from freely available spatial databases (Table S1 in Appendix S1). Study area boundaries were digitized and mean values for each variable were calculated for each study area, excluding open water and barren areas, which was up to 35% of the study area (mean  = 6.5%). For five study areas outside of our precipitation map, we used data from the nearest Environment Canada long-term weather records.We also derived a measure of topographic ruggedness [52], which we included as a covariate to index the increased land area associated with sloped areas. This variable was correlated to precipitation (*r* = 0.73, *N* = 90), but not to any other productivity variables (*r*<0.28, *N* = 90).

Landcover had been previously assigned into 3 structural classes at 500 m resolution: herbaceous (which includes shrubs <2 m in height), trees >2 m tall, and barren ([53]; Table S1 in Appendix S1). We surmised that in areas where the two bear species are sympatric, grizzly bears would monopolize resources in open areas and we used the proportion of each study area which was tree covered to index inter-specific interaction, while recognizing that the lack of tree canopy may also increase the vegetation resources available to bears. We calculated the proportion of forest in the vegetated portion of each study area by summing the proportion of pixels rated as >25% forest. We derived a measure of herb-shrub cover to index vegetative forage under the general assumption that forage is more abundant in non-forested areas. We calculated the proportion of herb-shrub area by summing the pixels with >50% herb-shrub cover. Pixels that were rated as 100% barren were excluded from spatial calculations (using a GIS mask); these areas were mostly rock and ice.

We used the fraction of salmon and animal tissue in the diet of bear populations as surrogates for salmon and terrestrial meat availability. Diet fractions may not be linearly related to resource availability, but deriving measures of salmon and meat availability across large areas was not feasible. Salmon and terrestrial meat components of the diet were predicted using isotope analysis of grizzly bear hair collected from each study area [54]. Where we did not have hair samples to estimate diet, we calculated mean values from spatial raster-based maps built from a continent-wide diet dataset ([54], digital data available in SOM). Unoccupied areas were necessarily assigned diet fractions in this way. We based diet fractions on direct feeding observations for two study areas (Nome and Midsu), because the model predictions suggested no salmon in the diet, and this was known to be incorrect [2]. For one area (Tweedsmuir), we recalculated diet using just the samples collected in the study area, because the map-derived values included areas where salmon were not available and were likely inaccurate. We also applied this value to the nearby Kimsquit-Dean area, because the mapped diet was likely biased low due to a drastic decline in salmon numbers in the nearby Owikeno Sound ([55] and unpublished data). Kokanee (*Oncorhynchus nerka*) were considered part of the salmon component of the diet. Kokanee diet fractions presented in Mowat and Heard [54] were reduced by half, based on further data collected from kokanee in central British Columbia (D. Heard, unpublished data). We also derived a categorical variable indexing the importance of salmon where 0 meant no salmon available, 1 for areas with little salmon, such as some interior areas, and 2 for those areas where bears were considered to derive most of their resources from salmon.

Human and livestock density was used to index human displacement, disturbance, and unreported bear mortality. We tested log transformations of these variables, because the influence was expected to be nonlinear [56]. The number of people and livestock (cattle and sheep) were calculated from polygon-based census data for the US (2000) and Canada (2001). Count data were used to calculate density for each census unit and density was used to calculate the number of resident people and livestock in each study area. In order to minimize the number of variables we added the human, cattle and sheep counts together to produce a single composite variable; this index combined both the various human impacts with the threat of direct mortality posed by livestock grazing.

Human-caused mortality necessarily reduced bear density and was entered directly and as a squared term to account for the non-linear influence of recent mortality on the standing population. We estimated the number of bears killed by people from government databases or published accounts. Counts of all legally killed bears have been recorded since at least the mid 1970s for all the jurisdictions in this study and represent a minimum number of bears killed by people. We calculated the annual kill rate (number bears killed/bear population estimate) over the 10 years previous to each density estimate. Unoccupied areas were assigned mean kill rates so these records did not bias the fitting of this variable. Raw data are available in Appendix S2.

### Statistical analysis

We used Principal Components Analysis (PCA; [57]) to contrast >3 variables that were surrogates for the same limiting factor. Principal component scores were considered, but not used, because of the difficulty in interpreting the resulting regression equation [58].

Ordinary least squares regression cannot be used directly to fit these models, because of the inclusion of study areas where no grizzly bears were located. For these points, the assumption of normally distributed errors about the regression line is violated. Consequently, Tobit regression [59] was used. In this model, a latent (hidden) variable (Y *) follows the ordinary linear model:
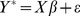



However, only the max(0, Y *) can be observed, i.e. it is impossible to observe negative densities.

Maximum likelihood was used to fit the Tobit model (Proc QLIM, SAS 9.2). Models were fit where every study area was given equal weight and where study areas were weighted by the inverse of the relative CLs to downweight study areas with less precise estimates of density.

We constructed an a-priori suite of models based on expert judgment, including variables thought to affect grizzly bear densities. Additional models were added to the initial model set where potential predictors were dropped or transformed.

Potential models included transformed values for human and livestock density to approximate the known form of the relationship, based on previous research as described earlier. We also included indicator variables that controlled for 3 cases we considered outliers, based on initial screening of the data. Two study areas had high and presumably unsustainable mortality rates, and one unoccupied area had very high human density. We used the small-sample corrected AIC_c_ to compare model fit, ranked models using AIC weights [60], and we examined top-ranked models for the presence of uninformative variables [61]. We investigated the leverage of each record in the top-fitting models using influence plots based on simple regression to check for individual study areas that may account for the inclusion of a predictor in the model. Residuals from the top-fitting models were examined for outliers and distribution [57].

We used a leave-one-out jackknife procedure to estimate the increase in prediction error that might occur when the model was used to predict densities outside of the set used to build the model. Each individual study area was sequentially dropped from the analysis, the remaining data were used to fit the model being examined, and this fitted model with sample size *n*-1 was then used to predict density for the study area held out.

## Results

**Figure 1:**
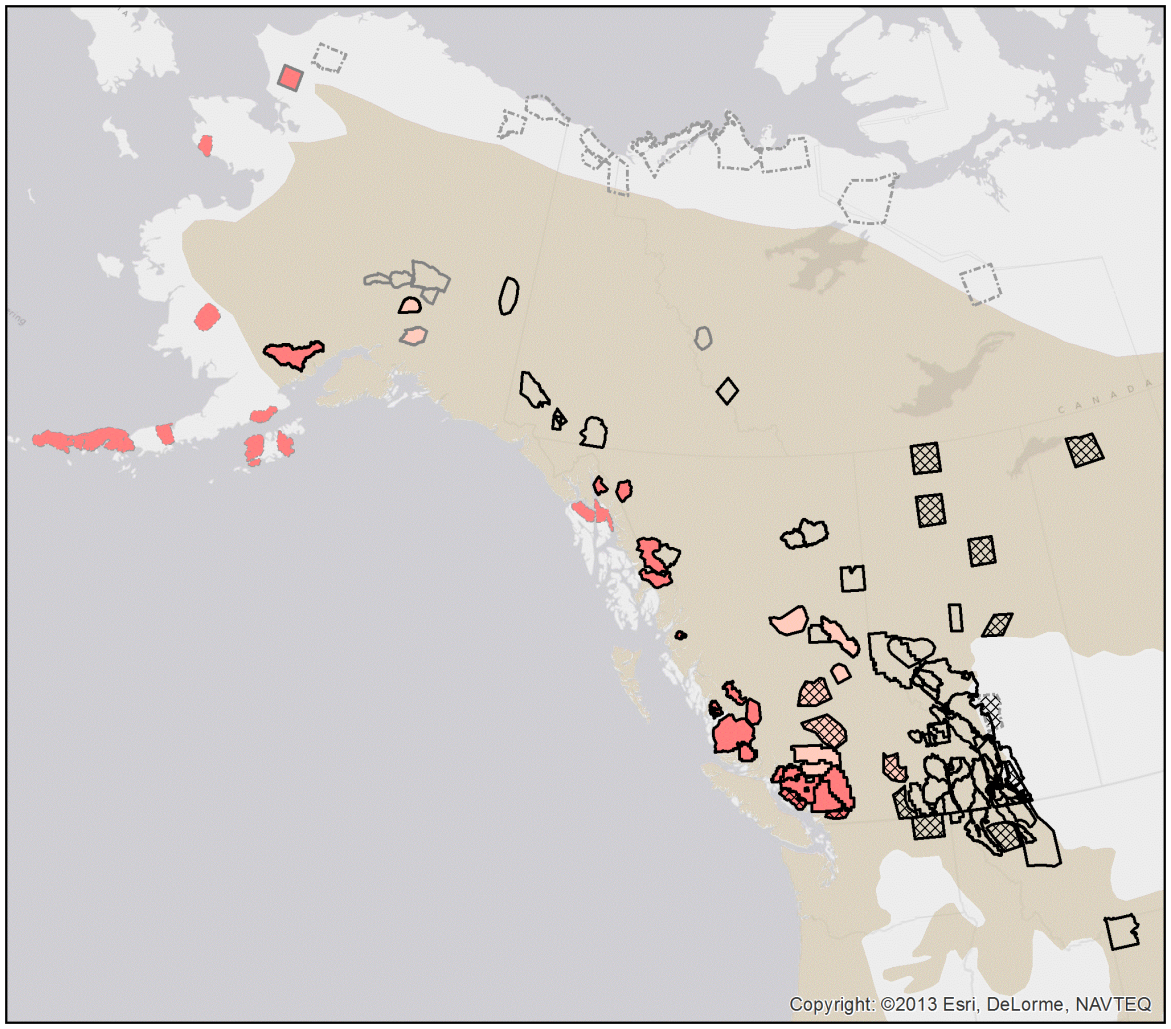
Estimates of grizzly bear density in North America. Areas currently unoccupied are filled with hatching. The presence of black bears throughout the study area is denoted by a black outline, partial presence of black bears by a gray outline and a hatched gray or no outline means black bears did not occur on the study area. Areas without salmon are not colour filled, those with abundant salmon are filled in red, and those where salmon were present but not abundant in rose. The black bear distribution in North America is shown in tan [91].

We derived 118 estimates of density including 16 for areas currently unoccupied by grizzly bears (Tables 2 and 3). This total included 2 repeated inventories for 6 study areas that were carried out in different years. The number and precision of those inventories was greatest in the southeastern portion of the range of grizzly bears and lowest in Yukon and the Northwest Territories (Figure 1). We do not believe there was a systematic bias in the data based on the selection of study areas because the motivation for inventories was not just conservation concern. Inventories were done for research, impact assessment, native land claims, ecological benchmarks and trend monitoring in National Parks.

**Table 2:** Descriptive statistics for data used to build models to predict grizzly bear density in interior North America.

Variable	Occupied areas					Unoccupied areas				
	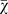	SD	Min	Max	*n*	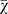	SD	Min	Max	*n*
Population size	92	96	15	765	76	0	0	0	0	14
Study area size (km^2^)	5110	4297	789	22875	76	6358	1629	3913	8959	14
Barren (% of study area)	6.7	8.6	0.0	34.6	76	1.7	2.1	0.1	6.7	14
Density (barren area removed)	23.0	15.1	2.5	64.6	76	0.0	0.0	0.0	0.0	14
CL relative (% of density)	1.0	0.5	0.1	1.9	76	0.8	0.3	0.5	1.0	14
Human-caused mortality (%)	3.7	3.9	0.0	20.1	76	0^1^	0.0	0	0	14
Annual precipitation (cm)	84	40	16	199	76	52	7	43	68	14
NDVI	116	15	77	137	76	129	5	115	136	14
AET	296	104	97	443	76	342	60	228	440	14
Average annual temperature	−4.0	5.0	−17	3.7	76	0.9	3.1	−5.0	4.7	14
Ruggedness	3.9	1.4	1.0	6.0	76	2.2	1.3	1.0	4.2	14
Trees (>25% per pixel)	48.1	34.7	0.0	99.5	76	69.7	30.0	8.4	99.7	14
Herb-shrub (>50% per pixel)	57.6	28.1	3.1	99.1	76	50.6	28.4	6.8	94.0	14
Salmon (% of diet)	1.0	3.0	0.0	14.0	76	1.5	3.6	0.0	10.2	14
Kokanee (% of diet)	0.7	3.5	0.0	26.0	76	0.6	1.5	0.0	5.1	14
Meat (% of diet)	25.1	15.5	0.0	58.2	76	29.3	13.9	12.5	48.1	14
Human density (humans/km^2^)	0.9	1.7	0.0	8.4	76	4.3	5.6	0.0	21.4	14
Livestock density (animals/km^2^)	1.7	5.5	0.0	39.4	76	11.5	16.9	0.0	53.2	14

Appendix S1 for detailed description of GIS derived variables. See Table S1 in

^1^ The kill rate in unoccupied areas was zero, but we used the mean rate of 3.7 for occupied areas during analysis so that these areas did not bias the distribution for this variable.

**Table 3:** Descriptive statistics for data used to predict grizzly bear density in coastal North America.

Variable	Black bears present					Black bears absent				
	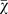	SD	Min	Max	*n*	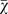	SD	Min	Max	*n*
Population size	81	88.6	0	352	17	619	450	102	1548	11
Study area size (km^2^)	3743	2858	431	9854	17	2829	2463	228	9163	11
Barren (% of study area)	16.0	10.4	0.7	43.4	17	9.6	10.0	1.1	35.7	11
Density (barren area removed)	31.1	25.9	0	86.6	17	332	215	37	856	11
CL relative (% of density)	1.1	0.5	0.1	2.0	17	0.6	0.5	0.2	1.5	11
Human-caused mortality (%)	2.3	2.6	0.0	10	17	3.8	2.7	0	7.3	11
Annual precipitation (cm)	275	111	115	473	17	160	57	101	255	11
NDVI	109	16	75	130	17	104	12	79	115	11
AET	370	58	263	474	17	321	30	239	351	11
Average annual temperature	1.6	2.5	−2.6	6.7	17	1.0	2.0	−4.5	2.6	11
Ruggedness	5.5	0.6	4.2	6.2	17	4.3	1.0	2.6	5.4	11
Trees (>25% per pixel)	54.4	23.1	17.8	96.8	17	40	25	1	79	11
Herb-shrub (>50% per pixel)	44.9	16.6	8.8	71.6	17	68	21	36	98	11
Salmon (% of diet)	41	22	0	78	17	59	15	28	82	11
Kokanee (% of diet)	0.4	0.8	0.0	3	17	0	0	0	0	11
Meat (% of diet)	2.3	3.9	0.0	13.4	17	3	11	0	36	11
Human density (humans/km^2^)	4.3	13.4	0.0	55.0	17	0.6	1.6	0.0	5.5	11
Livestock density (animals/km^2^)	1.1	4.3	0.0	17.9	17	0.01	0.00	0.0	0.02	11

Appendix S1 for detailed description of GIS derived variables. We separated areas where black bears were absent, because there were large difference in density between these areas. See Table S1 in

**Figure 2:**
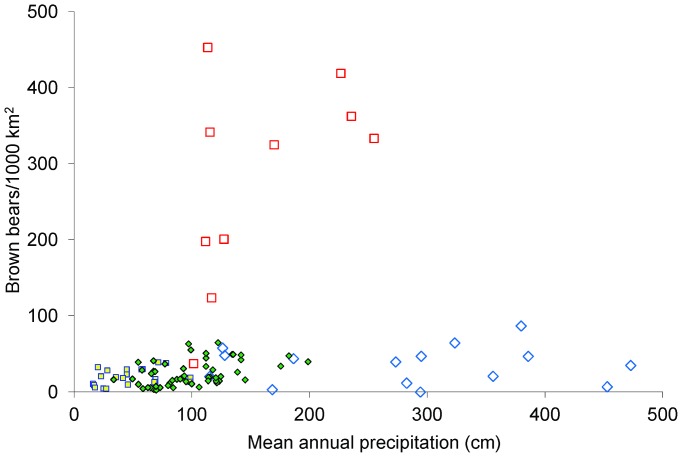
The relationship between grizzly bear density and mean annual precipitation. Study areas where grizzly bears were allopatric are denoted by squares and where black and brown bears were sympatric by diamonds. Open symbols denote coastal study areas where salmon was a major component of the diet; filled symbols show study areas where salmon were few. Unoccupied areas and one coastal area where brown bears were allopatric and at very high density (856) are not shown.

In coastal areas, where salmon were abundant (i.e., >27% of diet), grizzly bear density estimates were up to an order of magnitude higher than in interior areas (Figure 2). In addition, coastal grizzly bear density estimates were much higher in areas where black bears were absent than where they were present (Table 3). The one exception was the Kuskowim Delta in westcentral Alaska. This area had modest grizzly bear density and their diet contained more terrestrially derived meat (presumably caribou) than salmon or vegetation (C. Stricker, USGS, Denver, unpublished data). Coastal areas where black bears were present had much lower grizzly bear density, and the proportion of salmon in the grizzly bear diet was also more variable (Table 3). We concluded the availability of salmon led to fundamentally different ecological relationships so we built separate models for coastal and interior areas based on an arbitrary cut-off of 20% salmon in the diet. We further separated the coastal data into those areas where black bears were sympatric with grizzly bears and those where black bears were absent. We did not build a predictive model for coastal areas where black bears were absent because there were no areas like this in British Columbia.

In interior areas, density varied from 2.5 to 65 grizzly bears/1000 km^2^ and there was a broad range of values for most independent variables (Table 3). Human and livestock density was higher for unoccupied areas, presumably because humans were the ultimate cause of the extirpation in many of these areas.

**Figure 3:**
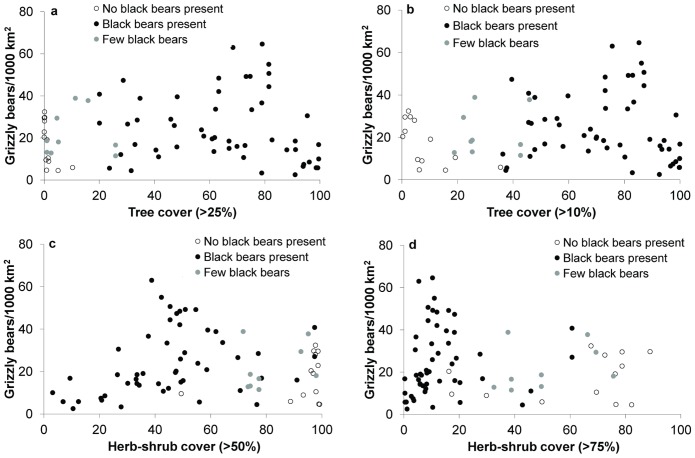
The relationship between vegetation cover and grizzly bear density. These are 76 sites from across interior North America where salmon is a minor component of the diet. We compare this relationship between 2 variables, tree cover (a–b) and herb-shrub cover (c–d). We also present 2 levels of summary within each study area for each variable. For example, tree>10% means that we summed the pixels where tree cover was >10% and calculated the proportion of the study area where this occurred. Black bears appear to be absent from areas where grizzlies are present and trees cover < about 20% of the study area. The proportion of the study with >25% tree cover appears to best describe this process.

In interior areas, the first eigenvector of a PCA suggested all 5 vegetation productivity variables were correlated and that ruggedness and NDVI contrasted in the second eigenvector. These results suggested ruggedness and NDVI should be included in the multivariate analysis, but that any of the five productivity variables could substitute for one another. For this reason we decided to include all five variables in the analysis. A second PCA with the vegetation type variables also demonstrated strong correlation among 4 of the 5 variables, whereas the fifth variable, herb100, was nearly invariant across the data. These results suggested that any one of the vegetation type variables could be used to index vegetation cover. We chose herb50, because we felt it would index bear food with the most sensitivity. We also included tree25 in the global model to index black bear competition, which was supported by comparing black bear presence across vegetation cover. Although the vegetation cover variables were strongly correlated, the presence of black bears was most clearly separated across the tree25 variable (Figure 3). A third PCA for the human influence variables showed essentially a single component that was an average of all 5 variables. Both human and livestock density showed a threshold with density, which suggested a non-linear relationship. We excluded the 2 variables that measured human and livestock density surrounding each study area, because there was no indication that density trended to zero at high human or livestock density [56].

There was very little variation in salmon in diet (which included kokanee) in interior areas and so the composite of fish and terrestrial meat in the diet was dominated by the meat component. For this reason the food composite variable was not considered further in the analysis.

In coastal areas with black bears, a PCA for the vegetation productivity variables yielded similar results to the interior dataset and we considered all five variables for the same reasons as above. We also chose to include herb50 and tree25, because these two variables may contrast herbaceous food abundance with competition with black bears. Human density was the only human influence variable that had substantial variation across the dataset and was the only variable we included. Diet was dominated by salmon and this was the only diet variable considered (Table 2). Expectedly, there were no coastal areas with low precipitation (Table 2).

### Tobit regression for interior areas

Based on biological considerations and the above investigation of the data, we included the following variables in our global regression: precipitation, NDVI, AET temperature, ruggedness, herb50, tree25, salmon-in-diet or salmon-presence, meat-in-diet, human density, livestock density or human + livestock density, human-caused mortality, and (human-caused mortality)^2^. Log transformations were also compared for the human-influence variables. When we compared the effect of salmon-in-diet versus the salmon-presence variable, only the salmon-presence variable remained consistently in the top models and we therefore excluded the salmon-in-diet variable.

When we compared the prediction strength of ‘human and livestock density’ versus the single composite variable, the composite variable was not included in the top-competing models. A log transformation did not improve the fit of the composite variable. This suggests the form of the relationship between human density and bear density differed from that of livestock density and bear density, so we dropped the summed variable, ‘human plus livestock density’.

One unoccupied area had double the human density of the next lowest value and was considered an outlier. This study area (Thompson) and the two other study areas (Upper Susitna and Swan Hills), where the number of grizzly bears killed by people was very high, were modeled using indicator variables in order to test their influence on fit. No models that included these indicator variables were included in the top models, suggesting these cases did not unduly leverage the analysis (Table 4). ‘Salmon-presence’ and ‘meat-in-diet’ were usually in the top models and the regression coefficient for the salmon variable was positive, whereas, surprisingly, that of the meat variable was negative.

**Table 4:** The top 10 model selection results for study areas in interior North America (n = 90) relating grizzly density to variables that were hypothesized to be functionally related to density.

Model number	Model description	AIC_c_	ΔAIC_c_	K	AIC_c_ weight
2	Prcp_NDVI_AET_H50_LHum_Live_Rug	978.9	0.0	9	0.17
3	Prcp_NDVI_AET_H50_T25_LHum_Live_Rug	979.0	0.1	10	0.16
4	Prcp_AET_H50_Meat_LHum_Live_Rug	979.1	0.3	9	0.15
5	Prcp_NDVI_AET_H50_T25_Meat_LHum_Live	980.1	1.2	10	0.09
6	Prcp_NDVI_AET_H50_T25_SP_Meat_LHum_Live_Rug	980.2	1.4	12	0.08
7	Prcp_NDVI_AET_H50_SP_Meat_LHum_Live_Rug	980.5	1.6	11	0.07
8	Prcp_AET_H50_SP_Meat_LHum_Live_Rug	981.2	2.3	10	0.05
9	Prcp_AET_H50_T25_Meat_LHum_Live_Rug	981.5	2.6	10	0.05
10	Prcp_NDVI_AET_H50_T25_SP_Meat_LHum_Live	982.6	3.7	11	0.03
11	Prcp_AET_H50_T25_SP_Meat_LHum_Live_Harv_Rug	982.8	3.9	12	0.02

=  precipitation, NDVI = normalized differential vegetation index, AET =  actual evapotranspiration, H50 =  herbaceous and shrub cover >50%, T25 =  tree cover >25%, Meat  =  terrestrial meat in diet, SP = presence of salmon in diet, LHum =  log human density, Live  =  livestock density, Harv  =  human-caused mortality, Rug  =  ruggedness. The top-ranked model was excluded from this list, because it contained 2 uninformative variables. Variables are: Prcp

The weighted Tobit models gave highest AIC weight (18%) to the global model, but this model had 9 variables, whereas the next model, that had only 7 variables, had similar weight (17%) and an AIC value that was only 0.3 higher than the global model. We chose to exclude the global model on the basis that it contained noise parameters and considered Model 2 to be our best model (Table 4). Model 2 included: annual precipitation (sign positive), annualized NDVI (+), average annual AET (+), the proportion of pixels in the study area with more than 50% herb-shrub coverage (+), log human density (−), livestock density (−), and ruggedness (+). The top 3 models had similar weight and differed by the inclusion of tree cover (−), the proportion of terrestrial meat (−) in the diet, and the exclusion of NDVI. Rankings of models were relatively consistent between the weighted and unweighted analysis and we present only the weighted analysis here.

**Figure 4:**
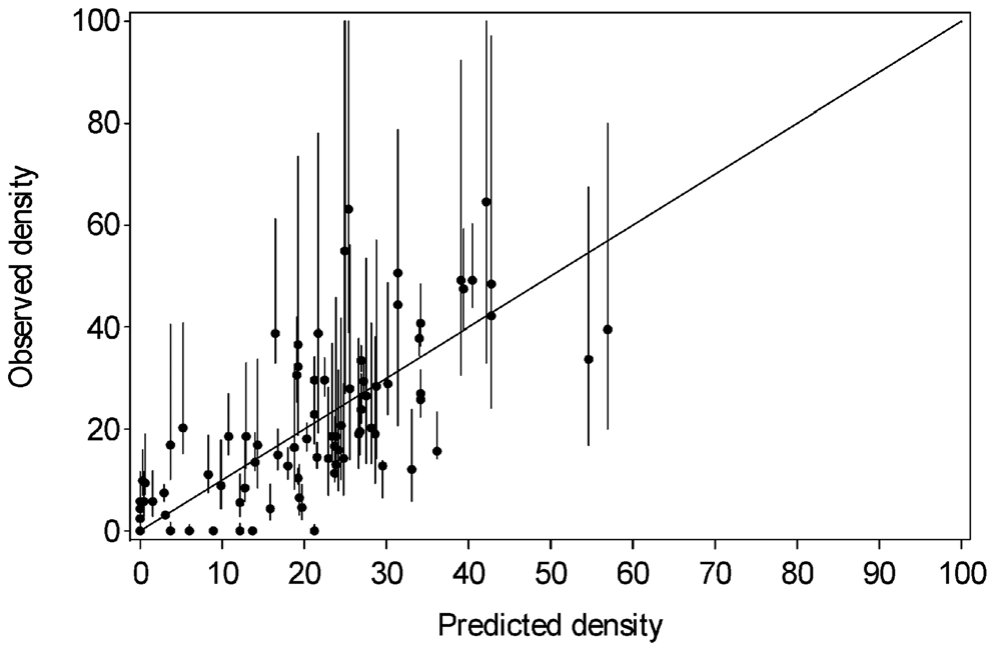
Observed versus predicted values of grizzly bear density (bears/1000 km^2^) using the best fit interior model described in Table 4. Data included 76 inventoried study areas and 14 unoccupied areas across the interior of western North America. Error bars are 95% confidence limits for observed data derived from the survey results or estimated subjectively, based on survey methods (see Methods for detailed description). The cases with the largest residuals often had the greatest error and were hence weighted lower in the regression.

Residual plots, using Model 2, showed no evidence that the regression assumptions were not met [57]. There was considerable variation in the residuals, but the largest residuals were associated with the observed densities with the widest CLs (Figure 4). For computational ease, ordinary least squares was used to compute approximate partial regression plots (leverage plots, [62]). These showed no serious outliers. The standard error of all predictions was approximately 10.5 bears/1000 km^2^. Two thirds of the CLs of the observed densities overlapped the 1∶1 line (Figure 4). Model errors were assumed to be independent among study areas. For 4 of the 90 study areas, the observed estimate fell outside the approximate 95% prediction interval for that site, but in all cases, the CL for the observed estimate overlapped the CL for the regression (Figure 4).

The mean model error for all jackknife runs was compared to the mean error of the model with all data included. For the top 10 interior models, the mean squared prediction error was 9–13% greater using the jackknife procedure compared to the model fit using all of the data. Model 1, the global model, which we excluded, had 12% higher model error using the jackknife procedure compared to the model error based on all the data whereas Model 2, our preferred model, had 9% higher error (Table S2 in Appendix S1). The increase in prediction error for the interior dataset was modest and indicated that predictions for study areas outside those used in fitting the model should be reliable. Model-averaged predictions and predictions from Model 2 were similar and further justified considering it our best model (Figure S1).

### Tobit regression for coastal areas

Based on biological considerations and the above investigation of the data, we included the following 7 variables in our global regression: precipitation, NDVI, AET, temperature, ruggedness, herb50, tree25, salmon-in-diet, and human density or log human density. The top model in the weighted Tobit analysis had 7 variables and the second model had 6 variables (Table 5). We excluded both models, based on the sample size to parameter ratio and because the third model included only 3 variables and the AIC value was only 2.2 higher than the top model, suggesting the other 4 variables in the top model were largely uninformative [61]. Our preferred model included the proportion of pixels in the study area with more than 25% tree cover (−), the proportion of salmon in the diet (+), and ruggedness (+). Rankings of models were relatively consistent between the weighted and unweighted analysis and we present only the weighted analysis here.

**Table 5:** The top 10 model selection results for study areas where grizzly and black bears were sympatric in coastal North America (n = 17) relating grizzly density to variables that were hypothesized to be functionally related to density.

Model number	Model description	AIC_c_	ΔAIC_c_	K	AIC_c_ weight
3	T25_salmon_Rug	157.7	0.0	5	0.17
4	Prcp_NDVI_AET_salmon_Hum_Rug	157.8	0.1	8	0.16
5	Prcp_NDVI_AET_salmon_LHum_Rug	158.0	0.3	8	0.15
6	Prcp_NDVI_Temp_H50_salmon_Hum_Rug	158.8	1.2	9	0.10
7	Prcp_NDVI_Temp_H50_salmon_LHum_Rug	159.9	2.2	9	0.06
8	Prcp_NDVI_Temp_H50_T25_salmon_Rug	160.2	2.6	9	0.05
9	Prcp_NDVI_Temp_T25_salmon_Hum	160.7	3.1	8	0.04
10	Prcp_NDVI_H50_T25_salmon_LHum_Rug	160.9	3.3	9	0.03
11	Prcp_NDVI_H50_T25_salmon_Hum_Rug	161.2	3.5	9	0.03
12	NDVI_T25_salmon_Rug	161.2	3.5	6	0.03

Table 4 for definition of variables; salmon  =  salmon in diet. The two top-ranked models were excluded from this list, because they contained four and three uninformative variables. See

The jackknife procedure was also used to compare among coastal models. The mean square prediction error from the jackknife models varied from 30–112% greater than that of the fitted model; it was 30% higher for Model 3, our preferred model (Table S3 in Appendix S1). Because the coastal model was based on a relatively small number of data points, compared to the number of predictors, Models 1 and 2 may have been fitting artifacts of the coastal study areas.

**Figure 5:**
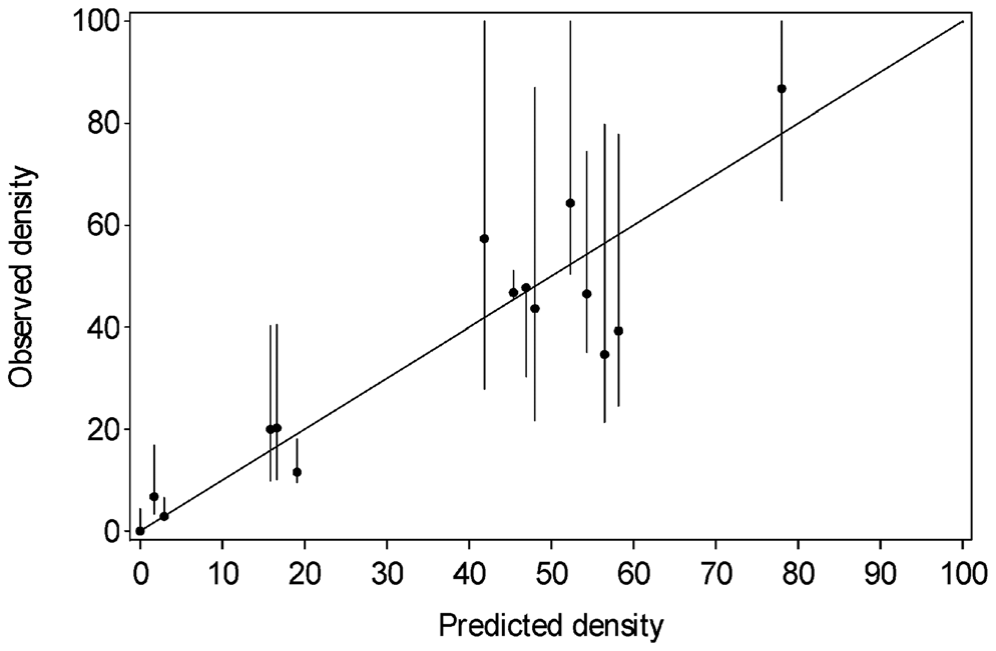
Observed versus predicted values of grizzly bear density (bears/1000 km^2^) using the best fit coastal model described in Table 5. Data included 15 inventoried study areas and 2 unoccupied areas across the interior of western North America. Error bars are 95% confidence limits for observed data derived from the survey results or, estimated subjectively based on survey methods (see Methods for detailed description).

Residual and leverage plots using Model 3 showed no evidence of lack of fit and no serious outliers. Plots of predicted versus observed densities showed a modest variation of residuals (Figure 5). Model averaged predictions and Model 3 predictions were similar, further justifying our use of Model 3 to predict density (Figure S2).

### Model predictions

Our models can be used to predict grizzly bear density for any area for which data exist for the input variables. Population size can be derived from density and these can be added to derive a population prediction for a larger area. We could not compute estimates of the uncertainty in composite predictions (i.e. for the total over several prediction areas), because predictions for study areas that are geographically adjacent with similar covariate sets are unlikely to be independent. Combining the uncertainties of the individual predictions will underestimate the uncertainty of the total. Without further information about the spatial structure of predictions from neighboring geographical areas, it is unclear how to compute an appropriate measure of uncertainty for the total over multiple study areas.

We predicted grizzly bear densities for Canada by summing the individual predictions within wildlife management units (Table 6). We used the interior Model 2 for all areas except coastal British Columbia where were used coastal Model 3 (Table S4 in Appendix S1)

**Table 6:** A summary of predicted numbers of grizzly bears in Canada and in National Parks by province, based on the coastal and interior models developed in this paper.

Province	Current population projection	Predicted population size from this paper	Prediction units	Number in National Parks
Alberta	867^a^	1250	ecoregions	396
British	16,014^b^	13,131	WMU's	126
Columbia		13,974	GBPU's	
		14,101	ecoregions	
Nunavut	1000^c^	8080	ecoregions	0
Northwest Territories	5100^c^	16,771	ecoregions	835
Yukon	6300^c^	10,404	guide territories	465
		10,465	ecoregions	

–5 WMUs), and territorial guide territory boundaries which were roughly similar to WMUs in size. We predicted density for small portions of each province using ecological unit mapping (ecoregions-the largest units used), provincial wildlife management units (WMUs), provincial grizzly bear population units (GBPUs, groups of 1

[94] with corrections for portions of the National Parks that were not included.^a^

b [92].

c [9].

Predicted grizzly bear densities in the Northwest Territories and Nunavut varied from zero in southcentral NWT to >30 bears/1000 km^2^ in parts of the western arctic coast, densities decline to the east and were much lower east of the Mackenzie River. In Yukon, the model predicted densities that vary from 0–31 bears/1000 km^2^ (Table S5 in Appendix S1). The lowest density was for the Labarge Unit surrounding the city of Whitehorse; the only place in the territory that had high human density. The Kluane area of southwest Yukon had higher precipitation than the range of data in the model (234 cm). This prediction may be biased high, if the relationship with density is not linear, but rather levels off beyond the range of the input data (Table 2). The average human-caused kill rate among population units was 1.3%, but was <1% when calculated for the entire territory. The Labarge unit had about 3 kills/year and predicted density of zero. This area may be a habitat sink for grizzly bears. The Dezadeash and Arkell units have mortality rates >5% and may be of conservation concern.

Predicted densities for British Columbia varied from 0 to 58 bears/1000 km^2^. The highest coastal densities were in northern areas where salmon consumption was high and tree cover was low. Interior areas with high density occurred throughout the province, but were always rugged areas with high rainfall and few people. Many units in the province were predicted to have low density and, whereas this was often associated with high human density, predicted densities in flat areas with low rainfall and low herb-shrub cover were also low. The average rate of human-caused mortality was 2.9%/yr, with most mortality from hunter kills (mean = 295/yr), but problem bear kills, and road and rail collisions, (mean = 61/yr) comprised a greater proportion of deaths in units with low predicted bear populations or relatively high human density (Table S4 in Appendix S1).

## Discussion

### Model Tests

We lacked the data to systematically test our models independently, so our testing was confined to comparisons with other approaches and examining areas of known low density. Boyce and Waller [10] used RSF models from previous research in Montana to predict potential abundance in the Bitterroot Mountains of central Idaho. This area is currently not occupied by grizzly bears so the number was meant to assess recovery potential. They had models for 3 seasons. The lowest predicted abundance was 321 bears and their highest seasonal prediction was 484 bears in spring. Our interior model predicted a total population of 657 bears (approximate 95% CL = 211–1103). Boyce and Waller [10] assumed that the population estimate from their two reference study areas were unbiased estimates of equilibrium density whereas bear numbers have increased in both areas since their research was done [63], [64].

Mattson and Merrill [65] provided an independent prediction of grizzly numbers in the Cabinat-Yak Recovery Area in Montana and Idaho, based on study area scale modeling of habitat capability and the depressive effects of human use. This area was one of our model areas and the 2004 population was estimated at 44 bears. The population was recovering from very low numbers and human-caused mortality appears to be limiting recovery [66]. Mattson and Merrill's model predicted the area could support 123 bears and our interior model predicted 130 bears. The current population size may be a result of top-down mortality forces and the predicted population sizes may be best viewed as potential population sizes if the population was released from top-down limitation.

### Predictions for extirpated zones and depressed populations as model tests

There were 14 study areas not known to support grizzly populations in the interior dataset. The predicted density was zero for 8 of these areas and >4 for the 6 other areas. Two areas had predicted density of >14 bears/km^2^. Grizzly bears do not occur in the southern boreal regions of NWT and Nunavut and northern Alberta and Saskatchewan [9]. This area is presumably grizzly bear-free naturally, because human density is very low. Four unoccupied study areas in this area had low human density and predicted grizzly bear densities were 0, 0, 9, & 21 (SE 10.6–10.8). Predicted grizzly density for ecoregions in the unoccupied area varied from 0 to 38 bears/1000 km^2^. The boreal portion of this unoccupied area was predicted to have low bear density, usually zero. The parkland and grassland areas south of the boreal zone were predicted to have zero bears throughout Alberta, but high densities were predicted in the same ecoregions in Saskatchewan. Human and livestock densities were much higher in Alberta, all other input data were similar. These results demonstrate that our interior model does not index the ecological factor or factors limiting distribution in all unoccupied areas well. Some combination of limiting factors excludes grizzlies from the Canadian prairies and surrounding boreal forest and this result was only correctly predicted by the interior model when human density or tree cover was high.

We also used our preferred models to predict equilibrium densities in known extirpated areas and zones with depressed populations that are designated as threatened or endangered in British Columbia and the lower 48 states ([8], [67] Table 7). Areas where grizzly bears are currently extirpated in British Columbia were all predicted to have densities <8/1000 km^2^ and <30 grizzly bears in the population unit. Most threatened units were also predicted to have small populations, but four units were predicted to have >150 bears (South Chilcotin Ranges, Squamish-Lillooet, Toba-Butte and the North Cascades). A recent inventory in the southern portion of the South Chilcotin Ranges unit suggests current populations may be similar to predicted numbers and hence no longer threatened [68]. The Squamish and Toba populations are recovering from human over-exploitation and are likely lower than predicted by the model. The North Cascades area in southwest BC and central Washington currently supports very few bears [69] although our model suggests the area may be capable of supporting several hundred bears on the Canadian side alone (Table 7). Previous habitat-based modeling suggested that the Canadian portion of the North Cascades could support 293 bears [70], which is similar to the 284 suggested by our model.

**Table 7:** Extrapolated grizzly bear densities and population sizes for a selection of areas in western North America that are currently unoccupied, occupied at low densities, or are considered threatened.

Population unit	Current population estimate	Predicted population size
Okanagan Valley, BC	0^a^	27
Thompson Valley, BC	0^a^	13
Caribou Plateau, BC	0^a^	0
Peace River agriculture zone, BC	<40^a^	29
North Cascades, BC	23^a^	284
Garibaldi-Pitt, BC	18^a^ or 0^b^	40
Squamish-Lilloet, BC	56^a^ or 52^b^	180
Toba-Bute, BC	75^a^ or >106^b^	211
Stein-Nahatlach, BC	61^a^ or 23^b^	129
South Chilicotin Ranges, BC	104^a^ or >147^b^	257
Blackwater West Chilicotin, BC	193^a^	24
Granby-Kettle, BC	81^a^	88
South Selkirks, BC	58^c^	85
Yahk, BC	20^c^	12
Bitteroot, ID and MT	0^d^	445
Cabinet-Yak, ID and MT	44^d^	130
North Cascades, WA	<5^d^	874
Northern Continental Divide, MT	765^e^	641
South Selkirks, ID and WA	30–40^c^	64
Yellowstone, WY, MT, ID	600^f^	567

Current population estimates were taken from government sources in British Columbia and the US and predicted population sizes were derived using our top coastal or interior model.

a [92].

b [68].

c [93].

d C. Servheen, USFWS, Montana, pers. com.

e [64].

f [67].

Our interior model predicted grizzly densities between 19 and 35 bears/1000 km^2^ in the six recovery areas in the lower 48 United States. This resulted in population predictions between 64 and 874 per study area, which is many more bears than currently occurs in two of these 6 areas (Table 7). As in Canada, the biggest discrepancy was in the North Cascades. Additionally, the Bitterroot Range is currently unoccupied, yet the interior model predicted 445 bears in this unit. In both these areas recovery is likely limited by the inability of grizzly bears to re-colonize these areas, not habitat characteristics.

We compared prediction units of various size and the results did not alter total population predictions for British Columbia or Yukon greatly (Table 6). The impacts were greater for British Columbia, because density was more variable. There are limits to the size of the area to which the model can be applied; ideally prediction units would be similar in size to the study areas used to build the model.

### Using the models to manage grizzly bear mortality

The models we developed, and the population sizes predicted from them, provide information to support the implementation of grizzly bear management policies. Population predictions were used to calculate human-caused mortality limits and predict habitat capability. Sustained yield management involves the trade-off between conservation risk and benefits to society. Conservation risk can be minimized by policy, such as reducing the maximum harvest rate, or by investment, such as by increasing inventory effort. In 1978 the Government of British Columbia began to move from seasonal hunting restrictions to a quota system to manage grizzly bear hunter kill. This change was effected in order to reduce conservation risk and indeed there is evidence some grizzly bear populations increased following to this policy change ([71] and G. Mowat, unpublished data), although other limiting factors may have changed as well. The paradox of these good intentions is that a quota system requires population sizes for every hunted population, because allowable kill is usually calculated as a portion of the standing population. Implementation of that policy required population estimates for all areas occupied by grizzly bears. This policy change precipitated a large increase in inventory investment [4], but population predictions were still required for many parts of the Province. The prediction method we describe here was another response to the change in harvest policy, and a reasonable balance between accuracy and investment. Our work used all currently available data, did not require expensive field testing, and provided predictions for all areas of British Columbia.

Our study demonstrates the uncertainty in extrapolating animal densities, even for species for which there is considerable inventory data and a good understanding of the population biology. Many areas were predicted to be unoccupied, or nearly so, when clearly this was not the case based on local knowledge or kill data, and vice-versa. This presents 3 problems for wildlife managers; 1) they will be forced to decide between those conflicting data (i.e., whether to allow hunting), 2) if they allow hunting then they will have to assign a density to the area either subjectively or using some other method, and 3) the credibility of the modeling process will be reduced, because it will be clearly evident that the model is ‘wrong’. These nuances and decisions are regularly confronted by wildlife managers and our example highlights the fact that removing subjectivity from the decision making process is impossible, even for very well studied species. Our study demonstrates the benefit of local knowledge, even for this highly data-driven management system. For example, 15% of our data were from extirpated areas where the population estimates and precision were based on local knowledge.

The population predictions were higher than current estimates for all Canadian provinces and territories except British Columbia [9]. Much of the discrepancy can be attributed to the fact the model predicted relatively high densities in northern boreal areas as discussed earlier. Densities predicted by the interior model in the tundra portions of the north are almost certainly too high in the eastern Arctic where large areas are not currently occupied or newly colonized. Population predictions for the Northwest Territories and Nunavut may be more realistic if these unoccupied areas were excluded from the prediction area.

Both our models predict more variable densities than other modeling efforts in British Columbia [5], [72] or Alberta [73]. If this variation is real, then earlier models were over-predicting density in some areas. Indeed, 12 management units in British Columbia appear to have annual kill rates higher than that allowed by policy (6%). A further 15 units that had predicted densities of zero had >2 reported bear kills annually during the past 8 years. This level of kill suggests a resident population. However, many of these units were predicted to have small populations. The above results could be due to model imprecision and mortality would need to be managed at larger scales to reduce this problem, as is currently done in BC. Management responses to this new information must occur on a unit by unit basis and incorporate other information such as local knowledge about distribution and movement among units; major food sources, such as salmon runs; hunter success; age and sex ratio of past kill; trend in kill numbers; and the trend and distribution of problem occurrences. We asked all regional population managers to evaluate the model predictions against all available data independent to the modeling process (Table 8). If the evaluation criteria suggested the model prediction was unrealistically low or high then we encouraged the manager to interpolate a density from a similar ecosystem. At the provincial scale, the mean predicted kill rate among wildlife units in British Columbia was 2.9% for those units that were predicted to support grizzly bears, which should be sustainable in most populations [50].

**Table 8:** Criteria used to evaluate individual model predictions that were independent of the model process.

Evaluation data required	Evaluation reasoning	Evaluation outcome
Anecdotal data including the locations where bears were sighted	If the distribution or number of bears people see is increasing this suggests an increasing population	Sightings of sows with cubs suggest the unit is occupied; distributional changes suggest corresponding changes in bear numbers; increased sightings suggest an increasing population
Locations where bears were killed or conflicted with people	The distribution of conflict or kill locations over time may suggest expanding, static or contracting bear distribution	Conflicts with sows and cubs suggest the unit is occupied; distributional changes suggest corresponding changes in bear numbers; increased conflicts suggest the population is increasing
Absolute ages of dead bears (from all human caused mortality)	Females older than 7 years are likely residents because they are unlikely to emigrate from their home range	Presence of resident bears suggests the unit is occupied; older median age at mortality of males suggests a lower kill rate
Age by sex of bears in the hunter kill	Trend in median age suggests a population trend	Decreasing age of males or increasing age of females may signal a declining population
Hunter success rates	Trend in success suggests a population trend	Higher success rates may indicate an increasing population
Proportion of females in the hunter kill	Trend in female proportion suggest a population trend	Increasing proportion of females suggests a declining male population

These criteria can be used to confirm residency, to identify suspect predictions, evaluate a predicted level of harvest, or help decide what level of harvest to allow.

### Biological implications of the models

We demonstrate that grizzly bear density is related to general indices of resources in the environment. Our results suggest that ultimate factors, such as vegetation biomass and productivity, vegetation structure, and protein abundance and availability, influence grizzly bear density across its North American range. We further show the degree to which density can be reduced by human influences, other than hunting, although the limiting effect of competition on density was equivocal. Other research has demonstrated the link between forage abundance and population growth in black bear [74], [75] and grizzly bear populations [28], [63], [76]. The negative effect of humans on grizzly bear population growth was empirically demonstrated in the latter two papers. Although the competitive effect of black bears on grizzly bear population density has been suggested [35] including potential individual and population level effects [77], [78] and, a potential mechanism described [42], demonstrating this effect on population density or growth remains elusive.

Our results suggest the plant portion of the bear diet is indexed by precipitation and NDVI. Precipitation likely indexes plant productivity whereas NDVI is thought to index plant biomass [79]. Ruggedness also appears related to density and, although it was correlated with precipitation (r = 0.73), the PCA analysis suggested these two variables contrasted in the third eigenvector, suggesting some level of independence. Ruggedness may index the increased surface area associated with sloped areas, which should provide greater plant biomass and increased variation in plant phenology among microclimatic sites [49]. A wide variety of studies have found grizzly bears select for more rugged terrain [11], [16], [33], [49]. Density was negatively related to forest cover and positively related to herb-shrub cover, presumably because bear plant foods are more abundant in non-forested areas. Habitat selection studies have demonstrated avoidance of forested areas by grizzly bears [33], [80]. However an alternative, and not exclusive hypothesis using our data, is that fewer trees may benefit grizzly bears by reducing competition with black bears. We could not isolate these two effects in our analysis.

In coastal areas density increased as the amount of salmon in the diet increased [28]. Our interior data also provide weak support for the generality of this observation, because the salmon presence variable appeared in 6 of the 10 top interior models and was always positively related to density, even though there was only a small range of variation in this variable. But, diet fractions did not necessarily correlate directly with salmon abundance or availability, especially where bears eat mostly salmon. In coastal areas where black bears were absent, salmon consumption was uniformly high (with one exception) and grizzly bear density was almost an order of magnitude higher than coastal areas where black bears were present (Table 3). Many observers have suggested that body size increases with dietary meat (reviewed in [54]) and although Mowat and Heard [54] showed that body size was strongly related to the amount of salmon in the diet, it was less correlated with terrestrial meat (see [28] for similar results). Regardless of the strength of the relationship between diet and body size, body size may be a better index of food quality or abundance than population density. In Alaska, bears in hunted areas were larger than bears in nearby ecologically similar areas that were not hunted ([47], see also [81]). We conclude that salmon availability influences grizzly bear abundance, but this relationship may be more complex than a simple linear relationship across the range of the species. Our data suggest a negative relationship between density and terrestrial meat availability, which may explain the observation by Mowat and Heard [54] above, but this observation may also be due to colinearity with other variables in our model.

Mattson and Merrill [56] and many others reported that human density negatively influences grizzly bear density and that grizzly bears cannot exist at a human density >7/km^2^. Our data generally support this hypothesis; only 1 of 101 occupied areas had human density >7/km^2^ and this study area straddled a heavily settled valley, but did not sample a great deal of the less settled mountains on either side of the valley. Mattson and Merrill [56] showed that the probability of grizzly bear persistence was inversely related to cattle density; similar to our findings that density was inversely related to livestock numbers.

Reported human-caused mortality explained relatively little of the variation in density when other factors were accounted for. Our data did not suggest a non-linear relationship between density and kill rate, as would be expected based on current theories of population growth [82], [83]. This was not too surprising given that most kill rates in our dataset were low. Low kill rates may cause little reduction in population size, especially when mostly males are killed. It has been suggested that grizzly bear populations show high compensatory responses only near ecological carrying capacity [84]. Hunter kill may also have been compensated for by immigration. Lags in the local impact of mortality due to immigration would confound comparisons of instantaneous measures of density and kill rate. Immigration sustained harvest has been observed in Eurasian brown bears where male-biased harvests may have indicated high immigration rates [85]. Similarly, populations that were reduced due to human-caused mortality, but have not recovered for various demographic reasons, would also confound the instantaneous comparison of density and kill rate, as discussed earlier. Human or livestock density may have accounted for some of the influence mortality had on density, but this was likely small, because correlations were weak between these variables in both datasets (r<0.07). Human and livestock density may be correlated with unreported human-caused mortality, however.

### Alternative methods for predicting grizzly bear density

Our approach to predicting density differs from most previous attempts that have usually been based on bear distribution or movement data and applied a use-versus-availability analysis approach [86]. This approach has been applied several times using radiotelemetry data from individual grizzly bears [10] or detection data [11], [65], [73], [80]. Although these studies used rigorous analysis techniques, often accounting for multiple scales and contrasting the importance of many different variables, the model structures appear unique to the landscape of origin and the authors were careful not to extrapolate the models much beyond the original study area which were roughly the size of one grizzly bear management unit in BC. Many models of this scale would be needed to predict density for BC. All final models included abstract landcover measures, such as elevation or greenness, that likely had complex relationships with other variables and with density, makings these types of models difficult to interpret in a functional sense.

In contrast we followed a more functional approach using measures of grizzly bear density at the landscape scale as the dependent variable rather the presence or abundance of individuals at a site. Density combines all the factors that influence population dynamics in a single measure and should be independent of factors such as individual behaviour which influence the outcome of finer scale analyses. Our analysis was unaffected by the relative abundance or availability of different resources within a study area that can limit predictions of an RSF model [17]. We consider the scale of our approach more appropriate than behavior-based models, because our dependent variable was measured at a similar scale to which we hope to make predictions [87] and, our model incorporated data across the entire area we intended to make predictions.

### Model weaknesses and improvements

Perhaps the largest weakness of our coastal model are the salmon diet data. This was a key variable, but it was extracted from a diet surface constructed from 81 diet measures across northwest North America, and many fewer along the coast. We augmented extracted data with local diet data where they were available, but this was sporadic. Perhaps the most important influence of the diet data on outcome was in deciding whether prediction areas were coastal or interior. The main information we used for this was the salmon diet data and when these were not available we used local knowledge though this was incomplete. Finer scale diet measures should improve the models and their application.

The variables driving our model were largely static. A dynamic model would require annual databases for each variable, such that every variable would be an appropriate multiannual mean pre-dating each survey. This would require vastly more digital data and the elimination of much early bear density data, because most databases begin in the 1980's or later. An effort of this level should also attempt to document human use of the landscape via a dynamic measure of road abundance and distribution; this too would require annual road layers for the past and the future. For its current application, it is crucial that local practitioners of the model understand its limitations so they understand where model predictions are least certain.

We were unable to index the availability of terrestrial meat and the correlation between precipitation and terrestrial meat in the diet (r = −0.63) undermined our ability to evaluate this factor using diet proportions. Also, the correlation of these two variables with tree cover (r_precip_ = 0.33, r_meat_ = −0.35) negated a controlled evaluation of the importance of competition with black bears (ignoring the question as to whether the tree cover variable was a reasonable index of competition). The availability of salmon was not correlated to other independent variables, but salmon was not a major contributor to diet in the interior. In the coastal model the salmon diet proportions were quite variable and appeared sensitive to changes in salmon availability [54], [55].

We were unable, at the scale we worked at, to index key vegetative foods like berries. Huckleberries (*Vaccinium membranaceum*) are a key food in most wetter environs and this resource is linked to natural burns [88]. We could not find data to index huckleberry abundance directly, nor could we find digital data that documented burn history at the scale of the continent.

Density estimates may vary with the size of the area over which they were measured [89], [90] but we believe we avoided this issue by selecting estimates based over large areas (Tables 3–4). There were weak negative relationships between density and area (*r*<−0.59) in all 3 datasets, but we believe this is explained by the need to have larger study areas in lower density populations in order to meet sample size requirements. The data we used were not subject to publication bias, a potential meta-analysis problem, because we used both published and unpublished data.

## Supporting Information

Appendix S1 **Supporting tables.**
(DOC)

Appendix S2 **Raw data.**
(XLS)

Figure S1 **Model averaging for the interior data.** Model-averaged predicted grizzly bear densities compared to predictions from the top model in Table 4. Data included 76 inventoried study areas and 14 unoccupied areas across the interior of western North America.
(TIF)

Figure S2 **Model averaging for the coastal data.** Model-averaged predicted grizzly bear densities compared to predictions from the top model in Table 5. Data included 15 inventoried study areas and 2 unoccupied areas across the interior of western North America.
(TIF)
