# Supplementary material for: Predicting Grizzly Bear Density in Western North America
Source: PLoS One. 2013 Dec 18;8(12):e82757. doi: 10.1371/journal.pone.0082757 (PMC3867401; doi:10.1371/journal.pone.0082757)
Supplement: Appendix S1 — Supporting tables. (DOC) [file pone.0082757.s001.doc]

**Table S1 Digital data preparation.** Variables extracted from digital databases that describe plant forage productivity and biomass and, human use of the landscape. We include a description of the original data used to build the GIS coverage and the spatial resolution (all data were shifted to raster format).

| Variable | Underlying data | Resolution (km) | Source for data | Reference |
| --- | --- | --- | --- | --- |
| mean annual precipitation | ground station weather data | 4 | PRISM  www.ocs.orst.edu/prism | Daly et al. 1994 |
| actual evapotranspiration (AET) | ground station weather data | 55 | UNEP/DEWA/GRID-Geneva  www.grid.unep.ch |  |
| normalized differential vegetation index (NDVI) | AVHRR satellites 1992-93 | 1 | EROS Data Center  Edcwww.cr.usgs.gov/landdaac/glcc | MTPE EOS Data Products Handbook Volume 1 |
| ruggedness |  | 1 | EROS Data Center  ftp://edcftp.cr.usgs.gov/ | Riley et al. 1999 [52] |
| human and livestock numbers | US and Canada census data | variable but 50-100 km outside of cities | Statistics Canada  US Censu Bureau  [www.statcan.ca](http://www.statcan.ca/)  www.census.gov/geo/www/tiger |  |
| Landcover: forest, water & barren (Vegetation Continuous Fields, VCF) | MODIS satellites | 0.5 | Global Landcover Facility, University of Maryland  Glcf.umiacs.umd.edu/data/modis/vcf | Hansen et al. 2003 [53] |

References

Daly, C, Neilson, RP, Philips, DL (1994) A statistical model for mapping climatological precipitation over mountainous terrain. Journal of Applied Meteorology 33: 140-158.

Hansen M, DeFries R, Townshend J, Carroll M, Dimiceli C, et al. (2003) Global percent tree cover at a spatial resolution of 500 meters: First results of the MODIS vegetation continuous fields algorithm. Earth Interactions 7: 1–15.

Riley SJ, DeGloria SD, Elliot R (1999) A terrain ruggedness index that quantifies topographic heterogeneity. Intermountain Journal of Sciences 5: 23–27.

**Table S2 Jackknife model comparison for the interior.** A comparison of the absolute model error and the mean error after removing a single observation iteratively using a jackknife procedure for grizzly bear densities in the interior of North America. The difference between the model error and the jackknife mean error tests for case sensitivity and helps identify over-fit models that may not generalize well. We chose model 1 as our top model in part because it had the lowest difference between the model error and the mean jackknife error.

| Model rank | Model description | Absolute model error | Mean jackknife model error | AICc model weight | Increase in prediction error^1^ |
| --- | --- | --- | --- | --- | --- |
| **1** | Prcp_NDVI_AET_H50_LHum_Live_Rug | 740 | 805 | 0.165 | 9% |
| **2** | Prcp_NDVI_AET_H50_T25_LHum_Live_Rug | 726 | 806 | 0.157 | 11% |
| **3** | Prcp_AET_H50_Meat_LHum_Live_Rug | 741 | 804 | 0.145 | 9% |
| **4** | Prcp_NDVI_AET_H50_T25_Meat_LHum_Live | 707 | 789 | 0.09 | 12% |
| **5** | Prcp_NDVI_AET_H50_T25_SP_Meat_LHum_Live_Rug | 711 | 808 | 0.084 | 14% |
| **6** | Prcp_NDVI_AET_H50_SP_Meat_LHum_Live_Rug | 731 | 815 | 0.074 | 11% |
| **7** | Prcp_AET_H50_SP_Meat_LHum_Live_Rug | 736 | 808 | 0.052 | 10% |
| **8** | Prcp_AET_H50_T25_Meat_LHum_Live_Rug | 737 | 815 | 0.045 | 11% |
| **9** | Prcp_NDVI_AET_H50_T25_SP_Meat_LHum_Live | 705 | 797 | 0.026 | 13% |
| **10** | Prcp_AET_H50_T25_SP_Meat_LHum_Live_Harv_Rug | 714 | 808 | 0.023 | 13% |

^1^ This is the increase in prediction error of jackknife model vs the full model.

**Table S3 Jackknife model comparison for the coast.** A comparison of the absolute model error and the mean error after removing a single observation iteratively using a jackknife procedure for grizzly bear densities in coastal North America. The difference between the model error and the jackknife mean error tests for case sensitivity and helps identify over-fit models which may not generalize well. We chose model 3 as our top model in part because it had the lowest difference between the model error and the mean jackknife error.

| Model rank | Model description | Absolute model error | Mean jackknife model error | AICc model weight | Increase in prediction error^1^ |
| --- | --- | --- | --- | --- | --- |
| **1** | Prcp_NDVI_AET_H50_T25_salmon_Rug | 52 | 110 | 0.286 | 112% |
| **2** | Prcp_NDVI_Temp_salmon_Hum_Rug | 68 | 141 | 0.157 | 107% |
| **3** | T25_salmon_Rug | 111 | 144 | 0.097 | 30% |
| **4** | Prcp_NDVI_AET_salmon_Hum_Rug | 72 | 133 | 0.09 | 85% |
| **5** | Prcp_NDVI_AET_salmon_LHum_Rug | 72 | 142 | 0.083 | 97% |
| **6** | Prcp_NDVI_Temp_H50_salmon_Hum_Rug | 47 | 90 | 0.054 | 91% |
| **7** | Prcp_NDVI_Temp_H50_salmon_LHum_Rug | 53 | 106 | 0.032 | 100% |
| **8** | Prcp_NDVI_Temp_H50_T25_salmon_Rug | 54 | 107 | 0.027 | 98% |
| **9** | Prcp_NDVI_Temp_T25_salmon_Hum | 72 | 131 | 0.021 | 82% |
| **10** | Prcp_NDVI_H50_T25_salmon_LHum_Rug | 57 | 115 | 0.019 | 102% |

^1^ This is the increase in prediction error of jackknife model vs the full model.

**Table S4 Grizzly bear densities for British Columbia.** Predicted population sizes for all wildlife management units in British Columbia known to support grizzly bears derived using our best fit coastal and interior model. The choice of which model to use was based on a survey of biologists known to be familiar with each area. Recent average annual kill rates (2007-2011) are presented based on these predicted densities.

| **Management unit** | **Predicted density** | **Predicted SD** | **Predicted LCL** | **Predicted UCL** | **Predicted population size** | **5 yr avg hunter kill** | **5 yr avg other kill** | **hunter kill rate** | **other kill rate** | **total kill rate** |
| --- | --- | --- | --- | --- | --- | --- | --- | --- | --- | --- |
| 1-14 | 14.6 | 8.9 | -3.2 | 32.4 | 85 | 16 | 0 | 2.3% | 0.0% | 2.3% |
| 1-15 | 24.2 | 8.3 | 7.7 | 40.8 | 141 | 28 | 2 | 2.5% | 0.2% | 2.7% |
| 2-5 | 4.1 | 9.1 | -14.0 | 22.3 | 12 | 0 | 0 | 0.0% | 0.0% | 0.0% |
| 2-6 | 36.8 | 7.6 | 21.7 | 51.9 | 80 | 0 | 0 | 0.0% | 0.0% | 0.0% |
| 2-7 | 8.9 | 8.4 | -7.9 | 25.6 | 8 | 0 | 0 | 0.0% | 0.0% | 0.0% |
| 2-8 | 0.0 | 9.0 | -22.9 | 13.2 | 0 | 0 | 0 | 0.0% | 0.0% | 0.0% |
| 2-9 | 20.5 | 7.9 | 4.8 | 36.3 | 32 | 0 | 0 | 0.0% | 0.0% | 0.0% |
| 2-10 | 14.1 | 8.0 | -2.0 | 30.2 | 15 | 0 | 0 | 0.0% | 0.0% | 0.0% |
| 2-11 | 40.9 | 11.1 | 15.9 | 60.4 | 109 | 0 | 1 | 0.0% | 0.1% | 0.1% |
| 2-12 | 0.0 | 9.4 | -21.1 | 16.4 | 0 | 0 | 0 | 0.0% | 0.0% | 0.0% |
| 2-13 | 46.6 | 7.6 | 31.3 | 61.9 | 108 | 0 | 0 | 0.0% | 0.0% | 0.0% |
| 2-14 | 51.7 | 7.7 | 36.3 | 67.1 | 103 | 0 | 2 | 0.0% | 0.2% | 0.2% |
| 2-15 | 47.4 | 7.6 | 32.2 | 62.6 | 113 | 0 | 0 | 0.0% | 0.0% | 0.0% |
| 2-18 | 0.0 | 9.2 | -25.6 | 11.1 | 0 | 0 | 0 | 0.0% | 0.0% | 0.0% |
| 2-19 | 0.0 | 9.4 | -34.4 | 3.3 | 0 | 0 | 0 | 0.0% | 0.0% | 0.0% |
| 3-15 | 10.6 | 8.1 | -5.6 | 26.9 | 21 | 0 | 1 | 0.0% | 0.6% | 0.6% |
| 3-16 | 31.4 | 10.5 | 8.4 | 50.4 | 92 | 0 | 1 | 0.0% | 0.1% | 0.1% |
| 3-32 | 16.8 | 10.5 | -3.9 | 38.1 | 60 | 0 | 0 | 0.0% | 0.0% | 0.0% |
| 3-33 | 22.5 | 10.5 | 2.9 | 44.8 | 45 | 0 | 0 | 0.0% | 0.0% | 0.0% |
| 3-34 | 22.1 | 10.7 | -5.3 | 37.5 | 11 | 0 | 2 | 0.0% | 2.3% | 2.3% |
| 3-35 | 35.7 | 10.6 | 8.0 | 50.4 | 30 | 4 | 5 | 1.7% | 2.1% | 3.8% |
| 3-36 | 23.1 | 10.5 | -1.1 | 40.7 | 62 | 7 | 0 | 1.4% | 0.0% | 1.4% |
| 3-37 | 8.7 | 10.5 | -12.7 | 29.3 | 15 | 0 | 0 | 0.0% | 0.0% | 0.0% |
| 3-38 | 2.9 | 10.6 | -19.2 | 23.2 | 5 | 0 | 0 | 0.0% | 0.0% | 0.0% |
| 3-39 | -1.1 | 10.6 | -20.0 | 22.4 | 0 | 0 | 0 | 0.0% | 0.0% | 0.0% |
| 3-40 | 16.9 | 10.6 | -6.9 | 35.3 | 28 | 0 | 0 | 0.0% | 0.0% | 0.0% |
| 3-41 | 21.4 | 10.5 | -2.4 | 39.5 | 20 | 0 | 0 | 0.0% | 0.0% | 0.0% |
| 3-42 | 33.1 | 10.5 | 8.6 | 50.6 | 49 | 1 | 0 | 0.3% | 0.0% | 0.3% |
| 3-43 | 41.5 | 10.6 | 16.8 | 59.3 | 42 | 1 | 0 | 0.3% | 0.0% | 0.3% |
| 3-44 | 46.4 | 10.6 | 21.4 | 63.8 | 75 | 6 | 1 | 1.0% | 0.2% | 1.2% |
| 3-45 | 29.3 | 10.6 | 9.9 | 52.3 | 61 | 0 | 0 | 0.0% | 0.0% | 0.0% |
| 3-46 | 26.2 | 10.5 | 8.3 | 50.2 | 75 | 0 | 0 | 0.0% | 0.0% | 0.0% |
| 4-1 | 47.2 | 10.8 | 23.5 | 66.6 | 75 | 11 | 2 | 1.8% | 0.3% | 2.2% |
| 4-2 | 32.3 | 10.5 | 10.4 | 52.2 | 40 | 14 | 1 | 4.4% | 0.3% | 4.7% |
| 4-3 | 1.4 | 10.6 | -18.9 | 23.4 | 2 | 0 | 3 | 0.0% | 17.0% | 17.0% |
| 4-4 | 8.4 | 10.6 | -15.2 | 27.1 | 9 | 0 | 3 | 0.0% | 4.0% | 4.0% |
| 4-5 | 10.3 | 10.6 | -12.8 | 29.4 | 9 | 0 | 1 | 0.0% | 1.4% | 1.4% |
| 4-6 | 16.2 | 10.5 | -7.4 | 34.6 | 36 | 1 | 4 | 0.3% | 1.4% | 1.7% |
| 4-7 | 22.5 | 10.5 | -0.3 | 41.7 | 42 | 0 | 1 | 0.0% | 0.3% | 0.3% |
| 4-8 | 20.1 | 10.5 | -3.2 | 38.8 | 43 | 0 | 3 | 0.0% | 0.9% | 0.9% |
| 4-9 | 10.4 | 10.6 | -12.1 | 30.3 | 12 | 0 | 1 | 0.0% | 1.0% | 1.0% |
| 4-14 | 13.0 | 10.5 | -8.5 | 33.5 | 12 | 0 | 4 | 0.0% | 4.3% | 4.3% |
| 4-15 | 22.3 | 10.5 | 0.2 | 42.1 | 38 | 2 | 1 | 0.7% | 0.3% | 1.0% |
| 4-16 | 28.4 | 10.5 | 7.2 | 49.2 | 50 | 0 | 0 | 0.0% | 0.0% | 0.0% |
| 4-17 | 32.5 | 10.5 | 9.8 | 51.7 | 54 | 13 | 0 | 3.0% | 0.0% | 3.0% |
| 4-18 | 27.0 | 10.5 | 4.4 | 46.5 | 37 | 5 | 2 | 1.7% | 0.7% | 2.4% |
| 4-19 | 36.6 | 10.5 | 12.3 | 54.4 | 52 | 3 | 0 | 0.7% | 0.0% | 0.7% |
| 4-20 | 20.9 | 10.5 | -1.2 | 40.7 | 79 | 13 | 8 | 2.0% | 1.3% | 3.3% |
| 4-21 | 24.2 | 10.4 | 3.8 | 45.5 | 32 | 10 | 2 | 4.0% | 0.8% | 4.7% |
| 4-22 | 27.9 | 10.5 | 7.1 | 49.0 | 66 | 15 | 24 | 2.9% | 4.6% | 7.4% |
| 4-23 | 24.2 | 10.6 | 1.9 | 44.2 | 81 | 49 | 24 | 7.6% | 3.7% | 11.3% |
| 4-24 | 33.0 | 10.6 | 11.6 | 53.9 | 60 | 13 | 2 | 2.7% | 0.4% | 3.1% |
| 4-25 | 22.9 | 10.4 | 1.3 | 43.1 | 70 | 15 | 3 | 2.7% | 0.5% | 3.2% |
| 4-26 | 22.8 | 10.5 | 0.4 | 42.5 | 67 | 14 | 3 | 2.6% | 0.6% | 3.2% |
| 4-27 | 39.1 | 10.5 | 17.5 | 59.5 | 54 | 24 | 0 | 5.5% | 0.0% | 5.5% |
| 4-28 | 49.7 | 10.6 | 27.3 | 69.5 | 37 | 14 | 0 | 4.7% | 0.0% | 4.7% |
| 4-29 | 47.2 | 10.5 | 23.4 | 65.6 | 38 | 11 | 0 | 3.6% | 0.0% | 3.6% |
| 4-30 | 40.3 | 10.5 | 18.0 | 59.9 | 51 | 9 | 4 | 2.2% | 1.0% | 3.2% |
| 4-31 | 36.0 | 10.5 | 14.7 | 56.6 | 46 | 10 | 0 | 2.7% | 0.0% | 2.7% |
| 4-32 | 27.8 | 10.4 | 6.9 | 48.6 | 69 | 13 | 1 | 2.3% | 0.2% | 2.5% |
| 4-33 | 49.9 | 10.7 | 24.8 | 67.6 | 105 | 29 | 0 | 3.5% | 0.0% | 3.5% |
| 4-34 | 26.1 | 10.5 | 5.0 | 46.9 | 98 | 25 | 5 | 3.2% | 0.6% | 3.8% |
| 4-35 | 18.2 | 10.5 | -5.1 | 37.1 | 46 | 12 | 2 | 3.3% | 0.5% | 3.8% |
| 4-36 | 25.9 | 10.4 | 8.2 | 49.8 | 92 | 27 | 2 | 3.7% | 0.3% | 3.9% |
| 4-37 | 49.3 | 10.7 | 27.9 | 70.7 | 92 | 20 | 0 | 2.7% | 0.0% | 2.7% |
| 4-38 | 47.7 | 10.8 | 21.7 | 64.9 | 173 | 48 | 1 | 3.5% | 0.1% | 3.5% |
| 4-39 | 45.4 | 10.6 | 21.0 | 63.5 | 109 | 30 | 1 | 3.4% | 0.1% | 3.6% |
| 4-40 | 36.7 | 10.5 | 18.3 | 60.4 | 77 | 27 | 0 | 4.4% | 0.0% | 4.4% |
| 5-3 | 0.1 | 10.7 | -19.0 | 23.8 | 0 | 0 | 0 | 0.0% | 0.0% | 0.0% |
| 5-4 | 5.7 | 10.6 | -12.2 | 30.2 | 43 | 0 | 7 | 0.0% | 2.1% | 2.1% |
| 5-5 | 15.0 | 10.5 | -3.6 | 38.5 | 79 | 0 | 2 | 0.0% | 0.3% | 0.3% |
| 5-6 | 21.6 | 10.5 | 4.2 | 46.1 | 104 | 0 | 0 | 0.0% | 0.0% | 0.0% |
| 5-7 | 23.3 | 8.0 | 7.4 | 39.2 | 142 | 0 | 4 | 0.0% | 0.4% | 0.4% |
| 5-8 | 1.9 | 8.8 | -15.7 | 19.6 | 15 | 38 | 48 | 31.2% | 39.4% | 70.5% |
| 5-9 | 13.6 | 8.0 | -2.5 | 29.6 | 102 | 13 | 3 | 1.6% | 0.4% | 2.0% |
| 5-10 | 18.8 | 10.6 | -3.3 | 39.0 | 63 | 0 | 0 | 0.0% | 0.0% | 0.0% |
| 5-11 | 29.0 | 10.5 | 9.0 | 50.9 | 53 | 0 | 3 | 0.0% | 0.7% | 0.7% |
| 5-12 | -3.5 | 10.8 | -20.9 | 22.4 | 0 | 0 | 15 | 0.0% | 0.0% | 0.0% |
| 5-13 | -7.3 | 10.8 | -26.0 | 17.1 | 0 | 0 | 1 | 0.0% | 0.0% | 0.0% |
| 5-15 | 23.1 | 10.6 | 6.7 | 49.0 | 209 | 69 | 4 | 4.1% | 0.2% | 4.4% |
| 5-16 | 28.0 | 10.6 | 11.7 | 54.2 | 30 | 0 | 0 | 0.0% | 0.0% | 0.0% |
| 6-1 | 4.6 | 10.8 | -14.0 | 29.1 | 24 | 1 | 2 | 0.5% | 1.0% | 1.5% |
| 6-2 | 0.0 | 10.8 | -4.6 | 38.7 | 0 | 8 | 1 | 0.0% | 0.0% | 0.0% |
| 6-3 | 2.3 | 8.9 | -15.5 | 20.0 | 35 | 18 | 20 | 6.4% | 7.1% | 13.5% |
| 6-4 | -2.5 | 10.6 | -19.6 | 22.7 | 0 | 12 | 1 | 0.0% | 0.0% | 0.0% |
| 6-5 | -14.6 | 10.9 | -35.8 | 7.9 | 0 | 0 | 0 | 0.0% | 0.0% | 0.0% |
| 6-6 | -10.0 | 10.8 | -28.1 | 15.0 | 0 | 1 | 0 | 0.0% | 0.0% | 0.0% |
| 6-7 | 36.6 | 10.6 | 14.8 | 57.1 | 154 | 20 | 1 | 1.6% | 0.1% | 1.7% |
| 6-8 | 1.4 | 10.5 | -18.0 | 24.0 | 13 | 23 | 14 | 22.6% | 13.8% | 36.4% |
| 6-9 | 17.0 | 10.6 | -4.4 | 38.0 | 225 | 31 | 12 | 1.7% | 0.7% | 2.4% |
| 6-10 | 58.2 | 8.0 | 42.2 | 74.1 | 74 | 0 | 0 | 0.0% | 0.0% | 0.0% |
| 6-11 | 0.0 | 10.9 | -43.7 | 0.1 | 0 | 27 | 11 | 0.0% | 0.0% | 0.0% |
| 6-14 | 20.3 | 8.3 | 3.7 | 36.9 | 180 | 14 | 17 | 1.0% | 1.2% | 2.2% |
| 6-15 | 30.9 | 7.7 | 15.6 | 46.3 | 250 | 22 | 10 | 1.1% | 0.5% | 1.6% |
| 6-16 | 7.5 | 9.1 | -10.7 | 25.6 | 37 | 32 | 3 | 10.9% | 1.0% | 11.9% |
| 6-17 | 46.4 | 10.8 | 26.5 | 69.8 | 461 | 64 | 2 | 1.7% | 0.1% | 1.8% |
| 6-18 | 0.0 | 10.8 | 24.6 | 67.7 | 0 | 25 | 1 | 0.0% | 0.0% | 0.0% |
| 6-19 | 31.3 | 10.5 | 5.6 | 47.6 | 234 | 21 | 2 | 1.1% | 0.1% | 1.2% |
| 6-20 | 32.2 | 10.4 | 8.9 | 50.6 | 432 | 74 | 5 | 2.1% | 0.1% | 2.3% |
| 6-21 | A |  |  |  | 426 | 46 | 6 | 1.3% | 0.2% | 1.5% |
| 6-22 | A |  |  |  | 310 | 31 | 5 | 1.3% | 0.2% | 1.5% |
| 6-23 | 15.5 | 10.4 | -8.8 | 32.9 | 167 | 25 | 8 | 1.9% | 0.6% | 2.5% |
| 6-24 | 19.3 | 10.4 | -5.2 | 36.5 | 179 | 26 | 3 | 1.8% | 0.2% | 2.0% |
| 6-25 | 15.5 | 10.4 | -5.5 | 36.0 | 243 | 51 | 5 | 2.6% | 0.3% | 2.9% |
| 6-26 | A |  |  |  | 490 | 64 | 1 | 1.6% | 0.0% | 1.7% |
| 6-27 | 25.5 | 10.8 | 7.6 | 50.8 | 113 | 17 | 1 | 1.9% | 0.1% | 2.0% |
| 6-28 | 29.1 | 10.9 | 8.7 | 52.3 | 71 | 16 | 1 | 2.8% | 0.2% | 3.0% |
| 6-29 | 55.8 | 9.8 | 36.2 | 75.4 | 223 | 24 | 0 | 1.3% | 0.0% | 1.3% |
| 6-30 | 21.0 | 10.4 | -1.7 | 39.9 | 140 | 21 | 7 | 1.9% | 0.6% | 2.5% |
| 7-1 | 29.1 | 10.6 | 4.7 | 47.0 | 56 | 0 | 0 | 0.0% | 0.0% | 0.0% |
| 7-2 | 30.7 | 10.5 | 6.9 | 48.9 | 92 | 25 | 0 | 3.4% | 0.0% | 3.4% |
| 7-3 | 34.9 | 10.5 | 9.3 | 51.2 | 129 | 43 | 0 | 4.2% | 0.0% | 4.2% |
| 7-4 | 26.4 | 10.5 | 4.2 | 46.2 | 60 | 14 | 0 | 2.9% | 0.0% | 2.9% |
| 7-5 | 26.5 | 10.4 | 5.2 | 46.9 | 77 | 32 | 1 | 5.2% | 0.2% | 5.3% |
| 7-6 | 22.8 | 10.7 | 5.7 | 48.4 | 46 | 14 | 1 | 3.8% | 0.3% | 4.1% |
| 7-7 | 1.8 | 10.6 | -17.7 | 24.9 | 5 | 14 | 6 | 33.0% | 14.2% | 47.2% |
| 7-8 | 2.5 | 10.7 | -14.9 | 27.9 | 5 | 0 | 0 | 0.0% | 0.0% | 0.0% |
| 7-9 | -3.5 | 10.7 | -22.4 | 20.2 | 0 | 0 | 1 | 0.0% | 0.0% | 0.0% |
| 7-10 | -7.7 | 10.7 | -27.1 | 15.8 | 0 | 0 | 0 | 0.0% | 0.0% | 0.0% |
| 7-11 | -10.3 | 10.9 | -29.4 | 14.1 | 0 | 0 | 1 | 0.0% | 0.0% | 0.0% |
| 7-12 | -11.8 | 10.8 | -32.1 | 11.2 | 0 | 1 | 4 | 0.0% | 0.0% | 0.0% |
| 7-13 | -16.1 | 10.9 | -35.8 | 7.7 | 0 | 0 | 12 | 0.0% | 0.0% | 0.0% |
| 7-14 | -14.1 | 10.9 | -34.4 | 9.1 | 0 | 0 | 1 | 0.0% | 0.0% | 0.0% |
| 7-15 | -15.9 | 11.0 | -36.5 | 7.5 | 0 | 0 | 3 | 0.0% | 0.0% | 0.0% |
| 7-16 | 9.5 | 10.6 | -12.6 | 29.8 | 36 | 11 | 1 | 3.9% | 0.4% | 4.2% |
| 7-17 | 23.8 | 10.5 | 1.3 | 43.2 | 64 | 20 | 1 | 3.9% | 0.2% | 4.1% |
| 7-18 | 48.9 | 10.7 | 24.7 | 67.7 | 181 | 40 | 0 | 2.8% | 0.0% | 2.8% |
| 7-19 | 18.1 | 10.6 | -4.4 | 38.0 | 79 | 25 | 1 | 4.0% | 0.2% | 4.1% |
| 7-20 | -6.4 | 10.7 | -24.6 | 18.2 | 0 | 13 | 4 | 0.0% | 0.0% | 0.0% |
| 7-21 | 8.8 | 10.5 | -10.9 | 31.1 | 59 | 24 | 4 | 5.1% | 0.9% | 6.0% |
| 7-22 | 16.4 | 10.5 | -5.9 | 36.1 | 68 | 14 | 3 | 2.6% | 0.6% | 3.1% |
| 7-23 | 33.8 | 10.6 | 9.8 | 52.1 | 146 | 33 | 1 | 2.8% | 0.1% | 2.9% |
| 7-24 | -1.4 | 10.7 | -22.7 | 19.9 | 0 | 6 | 1 | 0.0% | 0.0% | 0.0% |
| 7-25 | -16.1 | 10.8 | -31.0 | 12.1 | 0 | 6 | 0 | 0.0% | 0.0% | 0.0% |
| 7-26 | -3.4 | 10.7 | -23.2 | 19.6 | 0 | 11 | 1 | 0.0% | 0.0% | 0.0% |
| 7-27 | 6.9 | 10.5 | -10.6 | 31.3 | 37 | 24 | 2 | 8.2% | 0.7% | 8.9% |
| 7-28 | 5.1 | 10.7 | -15.4 | 27.2 | 39 | 17 | 1 | 5.5% | 0.3% | 5.8% |
| 7-29 | -4.1 | 10.7 | -21.2 | 21.5 | 0 | 10 | 0 | 0.0% | 0.0% | 0.0% |
| 7-30 | 5.6 | 10.6 | -15.1 | 27.5 | 13 | 4 | 7 | 3.8% | 6.6% | 10.4% |
| 7-31 | 10.8 | 10.5 | -11.2 | 30.8 | 58 | 23 | 0 | 4.9% | 0.0% | 4.9% |
| 7-35 | -7.1 | 10.7 | -26.8 | 15.8 | 0 | 7 | 1 | 0.0% | 0.0% | 0.0% |
| 7-36 | 10.9 | 10.7 | -10.5 | 32.2 | 36 | 12 | 3 | 4.1% | 1.0% | 5.1% |
| 7-37 | 28.4 | 10.7 | 7.3 | 50.1 | 177 | 58 | 1 | 4.1% | 0.1% | 4.2% |
| 7-38 | 24.0 | 10.6 | 2.8 | 45.2 | 308 | 54 | 1 | 2.2% | 0.0% | 2.2% |
| 7-39 | 43.1 | 10.7 | 21.5 | 64.3 | 370 | 40 | 1 | 1.3% | 0.0% | 1.4% |
| 7-40 | 29.3 | 10.4 | 6.8 | 48.5 | 216 | 20 | 1 | 1.2% | 0.1% | 1.2% |
| 7-41 | 27.5 | 10.5 | 6.6 | 48.7 | 208 | 54 | 0 | 3.3% | 0.0% | 3.3% |
| 7-42 | 20.7 | 10.7 | -0.7 | 42.1 | 120 | 15 | 13 | 1.6% | 1.4% | 2.9% |
| 7-43 | 8.2 | 10.6 | -14.2 | 28.3 | 25 | 14 | 2 | 7.1% | 1.0% | 8.1% |
| 7-44 | -4.5 | 10.6 | -25.6 | 16.9 | 0 | 9 | 2 | 0.0% | 0.0% | 0.0% |
| 7-45 | -4.0 | 10.6 | -23.8 | 18.8 | 0 | 9 | 1 | 0.0% | 0.0% | 0.0% |
| 7-46 | -6.2 | 10.8 | -25.2 | 17.9 | 0 | 0 | 1 | 0.0% | 0.0% | 0.0% |
| 7-47 | -4.4 | 10.9 | -24.6 | 19.1 | 0 | 1 | 1 | 0.0% | 0.0% | 0.0% |
| 7-48 | -4.1 | 10.7 | -24.7 | 18.1 | 0 | 14 | 1 | 0.0% | 0.0% | 0.0% |
| 7-49 | -6.6 | 10.6 | -26.6 | 15.9 | 0 | 26 | 2 | 0.0% | 0.0% | 0.0% |
| 7-50 | 22.3 | 10.6 | 1.3 | 43.8 | 137 | 13 | 23 | 1.2% | 2.1% | 3.3% |
| 7-51 | 21.9 | 10.5 | 0.4 | 42.6 | 381 | 91 | 2 | 3.0% | 0.1% | 3.0% |
| 7-52 | 26.6 | 10.6 | 1.5 | 43.8 | 459 | 71 | 6 | 1.9% | 0.2% | 2.1% |
| 7-53 | 3.7 | 10.7 | -15.1 | 27.8 | 37 | 16 | 2 | 5.5% | 0.7% | 6.1% |
| 7-54 | 9.6 | 10.7 | -10.0 | 32.7 | 71 | 24 | 3 | 4.2% | 0.5% | 4.8% |
| 7-55 | 1.7 | 10.9 | -16.2 | 27.3 | 30 | 0 | 0 | 0.0% | 0.0% | 0.0% |
| 7-56 | -1.5 | 10.8 | -19.8 | 23.5 | 0 | 0 | 0 | 0.0% | 0.0% | 0.0% |
| 7-57 | 21.0 | 10.6 | -1.0 | 41.4 | 52 | 11 | 3 | 2.6% | 0.7% | 3.3% |
| 7-58 | -0.2 | 10.6 | -21.1 | 21.3 | 0 | 0 | 2 | 0.0% | 0.0% | 0.0% |
| 8-3 | 7.1 | 10.7 | -15.5 | 27.3 | 9 | 0 | 0 | 0.0% | 0.0% | 0.0% |
| 8-4 | 6.8 | 10.6 | -15.5 | 26.9 | 7 | 0 | 0 | 0.0% | 0.0% | 0.0% |
| 8-13 | 14.3 | 10.5 | -7.0 | 34.8 | 8 | 0 | 0 | 0.0% | 0.0% | 0.0% |
| 8-14 | 9.6 | 10.5 | -11.3 | 30.9 | 24 | 0 | 2 | 0.0% | 1.0% | 1.0% |
| 8-15 | 11.5 | 10.5 | -10.5 | 31.6 | 32 | 0 | 0 | 0.0% | 0.0% | 0.0% |
| 8-23 | 12.8 | 10.5 | -10.2 | 31.7 | 33 | 1 | 3 | 0.4% | 1.1% | 1.5% |
| 8-24 | 20.7 | 10.4 | -3.2 | 38.5 | 26 | 1 | 2 | 0.5% | 0.9% | 1.4% |
| 8-25 | -1.2 | 10.7 | -24.7 | 18.1 | 0 | 0 | 0 | 0.0% | 0.0% | 0.0% |

^A^ These units were predicted using both the interior and coastal models by splitting the units based on salmon presence and then summing the predicted population sizes.

**Table S5 Predicted grizzly bear densities for Yukon.** Population sizes for all management units in Yukon Territory, Canada using our best fit model for interior areas. Grizzly bears are not known to rely heavily on salmon in any of these areas, although local feeding on salmon, arctic char, and marine mammals has been documented. Recent kill rates were based on predicted densities.

| NAME | Area | Predicted density | LCL | UCL | Population size | Human caused kill 1995-2004 | Kill rate |
| --- | --- | --- | --- | --- | --- | --- | --- |
| Aishihik | 22,898 | 19.1 | 1.0 | 43.2 | 436 | 83 | 1.9% |
| Anvil | 9776 | 24.9 | 3.8 | 45.9 | 243 | 20 | 0.8% |
| Arkell | 7537 | 9.2 | -12.7 | 29.3 | 69 | 41 | 5.9% |
| Big Salmon | 13,115 | 17.3 | -6.0 | 35.7 | 227 | 35 | 1.5% |
| Bonnet Plume | 10,664 | 30.7 | 7.8 | 50.3 | 328 | 46 | 1.4% |
| Cassiar | 35,572 | 27.1 | 3.2 | 44.9 | 965 | 45 | 0.5% |
| Dezedeash | 4564 | 17.6 | -2.4 | 39.4 | 80 | 48 | 6.0% |
| Eagle Plains | 22,736 | 29.7 | 7.9 | 50.3 | 676 | 1 | 0.0% |
| East Arctic | 9372 | 19.5 | -1.1 | 41.1 | 182 | 4 | 0.2% |
| Frances | 13,149 | 10.9 | -10.3 | 31.3 | 144 | 8 | 0.6% |
| Glenlyon | 9851 | 12.4 | -6.5 | 35.4 | 122 | 35 | 2.9% |
| Gold | 36,448 | 19.1 | -0.2 | 41.8 | 696 | 36 | 0.5% |
| Hart | 17,974 | 30.9 | 9.2 | 51.4 | 556 | 31 | 0.6% |
| Hyland | 28,359 | 16.1 | -3.3 | 39.0 | 457 | 27 | 0.6% |
| Klondike | 9669 | 30.4 | 9.0 | 51.0 | 294 | 31 | 1.1% |
| Kluane | 11,239 | 25.6 | 3.4 | 55.7 | 287 | 18 | 0.6% |
| Knorr | 10,294 | 28.9 | 5.7 | 47.7 | 298 | 1 | 0.0% |
| Laberge | 8308 | 0.0 | -22.9 | 19.6 | 0 | 29 | 0.0% |
| MacMillan | 21,781 | 30.4 | 6.4 | 48.0 | 661 | 48 | 0.7% |
| Nadaleen | 8257 | 31.4 | 9.0 | 51.4 | 259 | 46 | 1.8% |
| Nisling | 8883 | 21.8 | 3.9 | 46.9 | 194 | 43 | 2.2% |
| North Ogilvie | 14,863 | 30.5 | 9.6 | 51.9 | 453 | 40 | 0.9% |
| Old Crow Flats | 22,926 | 21.0 | -0.7 | 41.1 | 481 | 19 | 0.4% |
| Pelly | 15,296 | 23.5 | 0.5 | 42.2 | 359 | 39 | 1.1% |
| Richardson | 17,862 | 28.8 | 4.1 | 46.1 | 515 | 5 | 0.1% |
| Ruby | 2079 | 26.4 | 8.8 | 52.1 | 55 | 8 | 1.5% |
| Southern Lakes | 7282 | 8.5 | -12.0 | 30.0 | 62 | 25 | 4.0% |
| Stewart | 19,603 | 28.8 | 6.8 | 48.4 | 565 | 60 | 1.1% |
| West Arctic | 13,878 | 16.9 | -1.9 | 40.5 | 234 | 1 | 0.0% |
| West Ogilvie | 14,361 | 26.6 | 7.0 | 49.0 | 382 | 51 | 1.3% |
| White | 7311 | 16.4 | -0.6 | 41.7 | 120 | 20 | 1.7% |
| TOTAL |  |  |  |  | 10,404 | 944 |  |
